# Target-Specific Effects of Deep Brain Stimulation for Tourette Syndrome: A Systematic Review and Meta-Analysis

**DOI:** 10.3389/fneur.2021.769275

**Published:** 2021-10-20

**Authors:** Laura Wehmeyer, Thomas Schüller, Jana Kiess, Petra Heiden, Veerle Visser-Vandewalle, Juan Carlos Baldermann, Pablo Andrade

**Affiliations:** ^1^Faculty of Medicine and University Hospital Cologne, Department of Stereotactic and Functional Neurosurgery, University of Cologne, Cologne, Germany; ^2^Faculty of Medicine and University Hospital Cologne, Department of Psychiatry and Psychotherapy, University of Cologne, Cologne, Germany; ^3^Faculty of Medicine and University Hospital Cologne, Department of Neurology, University of Cologne, Cologne, Germany

**Keywords:** Tourette syndrome, tic disorders, deep brain stimulation, DBS, neuromodulation, systematic review, meta-analysis

## Abstract

**Background:** Extended research has pointed to the efficacy of deep brain stimulation (DBS) in treatment of patients with treatment-refractory Tourette syndrome (TS). The four most commonly used DBS targets for TS include the centromedian nucleus–nucleus ventrooralis internus (CM-Voi) and the centromedian nucleus–parafascicular (CM-Pf) complexes of the thalamus, and the posteroventrolateral (pvIGPi) and the anteromedial portion of the globus pallidus internus (amGPi). Differences and commonalities between those targets need to be compared systematically.

**Objective:** Therefore, we evaluated whether DBS is effective in reducing TS symptoms and target-specific differences.

**Methods:** A PubMed literature search was conducted according to the PRISMA guidelines. Eligible literature was used to conduct a systematic review and meta-analysis.

**Results:** In total, 65 studies with 376 patients were included. Overall, Yale Global Tic Severity Scale (YGTSS) scores were reduced by more than 50 in 69% of the patients. DBS also resulted in significant reductions of secondary outcome measures, including the total YGTSS, modified Rush Video-Based Tic Rating Scale (mRVRS), Yale-Brown Obsessive Compulsive Scale (YBOCS), and Becks Depression Inventory (BDI). All targets resulted in significant reductions of YGTSS scores and, with the exception of the CM-Pf, also in reduced YBOCS scores. Interestingly, DBS of pallidal targets showed increased YGTSS and YBOCS reductions compared to thalamic targets. Also, the meta-analysis including six randomized controlled and double-blinded trials demonstrated clinical efficacy of DBS for TS, that remained significant for GPi but not thalamic stimulation in two separate meta-analyses.

**Conclusion:** We conclude that DBS is a clinically effective treatment option for patients with treatment-refractory TS, with all targets showing comparable improvement rates. Future research might focus on personalized and symptom-specific target selection.

## Introduction

Tourette syndrome (TS) is a neurodevelopmental disorder characterized by motor and vocal tics. Tics have an onset in childhood and reach their peak between 10 and 12 years of age (1). A majority of patients experience reduced symptoms by late adolescence or early adulthood. Nevertheless, around 20% of patients continue to experience persistent, distressing, and even painful tics throughout adulthood (2). Tics can have a great influence on the patient's overall health and well-being, as they may disrupt daily functioning and adversely affect the quality of life (3, 4). The pathophysiology of TS is related to disturbances of a complex neural network with dysregulations of the cortico-basal ganglia-thalamo-cortical (CBGTC) circuits being of predominant importance (5–9). The sensorimotor circuit, but also the limbic and associative circuits are implicated in the heterogenous pathophysiology of TS (5, 10–12). Therefore, TS is in many cases accompanied by comorbidities such as attention-deficit hyperactivity disorder (ADHD), obsessive-compulsive disorder (OCD), or depression (13, 14). Importantly, comorbid disorders are associated with increased social problems and reduced quality of life (15). Conventional treatment approaches for TS include pharmacological and behavioral therapy that are beneficial for a majority of patients (16–19). Nonetheless, some patients do not respond to these treatments and remain severely affected. An alternative and safe treatment option for those treatment-refractory patients constitutes deep brain stimulation (DBS) (20).

In 1999, DBS for TS was introduced by Vandewalle et al. (21). The original target chosen by this group was the centromedian nucleus-substantia periventricularis-nucleus ventro-oralis internus complex (CM-Spv-Voi), informed by the experiences of Hassler and Dieckmann (22) with stereotactic thalamic lesions in this region. Thereafter, different targets have been selected based on the involvement of the CBGTC-circuits in TS pathophysiology. The most commonly used targets for TS include different thalamic nuclei and the globus pallidus internus (GPi). Within the thalamus, the centromedian nucleus–nucleus ventrooralis internus (CM-Voi) and the centromedian nucleus–parafascicular (CM-Pf) complexes have been used most frequently. This was motivated by their diverse connections to subcortical and cortical regions, including motor, associative, and limbic areas (23–25). The GPi consists of an anteromedial part (amGPi), which is densely connected with associative and limbic networks, and a posteroventrolateral part (pvlGPi), which mainly projects to sensorimotor areas (26, 27). Based on this differentiation, it can be assumed that the pvlGPi may be particularly effective in reducing tic symptoms, while the amGPi might be especially effective for the treatment of comorbid OCD symptoms (28–32). The selection of an ideal target for TS treatment is still a matter of debate and differences regarding clinical relevance remain unclear (33–38). Beyond that, target selection is complicated by the fact that the mechanism of action of DBS is still not fully understood, although, there is a growing consensus among researchers that DBS may exert its therapeutic effects by modulating the activity of widespread networks (20, 39–41). To date, the target choice is often a matter of preference of the centers, based on their surgical experience (42). On the contrary, some researchers have emphasized the idea that target selection should ideally be based on the individual characteristics of each patient. Hence, the patient's individual symptomatology and possible comorbid disorders should be taken into account in order to decide on the most appropriate target (34, 43).

Our objective was to examine the clinical effects of DBS for TS treatment with a systematic review and meta-analyses. First, we aimed to evaluate whether DBS is capable of reducing TS symptoms in the long-term. Our second goal was to evaluate whether the most commonly used targets, namely the CM-Voi, CM-Pf, the amGPi, and the pvlGPi, lead to different clinical outcomes regarding tic reduction and comorbid OCD symptoms.

## Methods

### Systematic Literature Search

A systematic literature search was conducted following the guidelines of Preferred Items for Reporting Systematic Reviews and Meta-analyses (PRISMA) (44). A search of the electronic database of PubMed was performed to identify the existing literature investigating the effects of DBS in TS patients. The search terms included “Tourette syndrome OR Gilles de la Tourette syndrome OR Tourette's disorder OR Tic disorder” AND “Deep Brain Stimulation OR DBS.” Literature search was narrowed to all available articles published from January 1st 1999 to July 8th 2021. Additionally, two recently published meta-analyses of Baldermann et al. (36) and Xu et al. (38) were screened for additional research articles. In order to be included, studies were required to meet the following conditions: (1) case report, case series, clinical trial, or randomized controlled study of DBS for patients diagnosed with TS or a tic disorder; (2) original, published and peer-reviewed; (3) written in English. Studies were excluded if (1) clinical data of the patients could not be identified, (2) the clinical outcome was not assessed by the Yale Global Tic Severity Scale (YGTSS), or (3) patients had already been described in other articles. Titles and abstracts in each study from the search results were independently screened for eligibility by two researchers (LW and JK).

### Data Extraction

The full text of the screened articles was further checked for eligibility and compliance with selection criteria by two researchers (LW and JK). If necessary, exclusion of duplicates was ensured by screening the patient demographics in the studies. Then, the following data were extracted from all studies included in the quantitative synthesis: first author name and publication year, number of participants, sex, age at surgery, DBS targets, follow-up (FU) range, pre- and post-surgery scores of the global YGTSS, total YGTSS, modified Rush Video-Based Tic Rating Scale (mRVRS), Yale-Brown Obsessive Compulsive Scale (YBOCS), and Becks Depression Inventory (BDI). When possible, individual patient data was gathered from the constituent studies. If two targets were evaluated in one patient, an additional case was added.

### Study Quality Assessment

The quality of each study was assessed using the classifications scheme developed by French and Gronseth (45). This scheme includes 4 levels of evidence, with level 1 representing high-quality studies with low risk of bias and level 4 representing studies with a very high risk of bias. Additionally, the quality of randomized trials was assessed using the Cochrane risk of bias tool for randomized controlled trials (46). Two researchers independently evaluated the risk of bias of each study (LW and JK).

### Statistical Analysis

The global YGTSS score (tic severity + impairment; range: 0–100, highest score representing worst clinical condition) served as primary outcome measure. Secondary tic-related outcome measures included the YGTSS total tic score (tic severity; range: 0–50), as well as the mRVRS. Additional secondary outcome measures included YBOCS and BDI assessments. Cases were weighted by the number of participants included in each individual study. Pre- and post-surgery primary outcome scores were compared using Wilcoxon signed-rank tests. Global YGTSS scores for maximum follow-up as well as for different postoperative time points (T1: ≤ 6 months; T2: ≤ 12 months; T3: >12 months) were compared with baseline scores (T0) across the whole sample. To examine whether YGTSS scores differed for the various postoperative time points Friedman's test was applied. In case of a significant result, *post-hoc* Dunn tests were conducted and Bonferroni-corrected for multiple comparisons. Regarding the secondary outcome measures, last reported YGTSS total tic, mRVRS, YBOCS and BDI scores were compared with preoperative baseline scores using Wilcoxon signed-rank tests. Subgroup analyses of YGTSS percentage change scores at T2 (6–12 months) were performed using Kruskal-Wallis tests in order to compare the four targets (CM-Pf, CM-Voi, amGPi, and pvlGPi). T2 was chosen as time point for the subgroup analysis because of its clinical relevance and temporal precision compared to T3 and maximum follow-up. *Post-hoc* pairwise comparisons using the Dunn-Bonferroni approach were performed in the case of significant results. Furthermore, absolute change scores of the YBOCS at maximum follow-up were compared between the four targets using Kruskal-Wallis tests. For the YBOCS scores, maximum follow-up was chosen as time point for the subgroup analysis, because a temporal categorization was not possible due to insufficient data. Again, *post-hoc* Dunn tests were performed in case of significant results and Bonferroni-corrected for multiple comparisons. Of note, articles were excluded from subgroup analyses if the target was not appropriately specified, or multiple targets were used and outcomes combined. Beyond that, three separate meta-analyses of randomized controlled and double-blinded trials (RCTs) were conducted with the YGTSS total tic score as primary outcome measure. A first meta-analysis was performed to examine the general effect of DBS across all targets. In addition, two separate meta-analyses were conducted including RCTs targeting the thalamus and GPi, respectively. Standardized means of the YGTSS total tic score were compared between the experimental condition (DBS ON) vs. control condition (DBS OFF). A random-effect model was used to account for heterogeneity among studies. Analyses were performed with SPSS 27 and the Review Manager 5.4.1. (47, 48). Significance levels were set at *p* < 0.05.

## Results

### Study Selection

**Figure 1:**
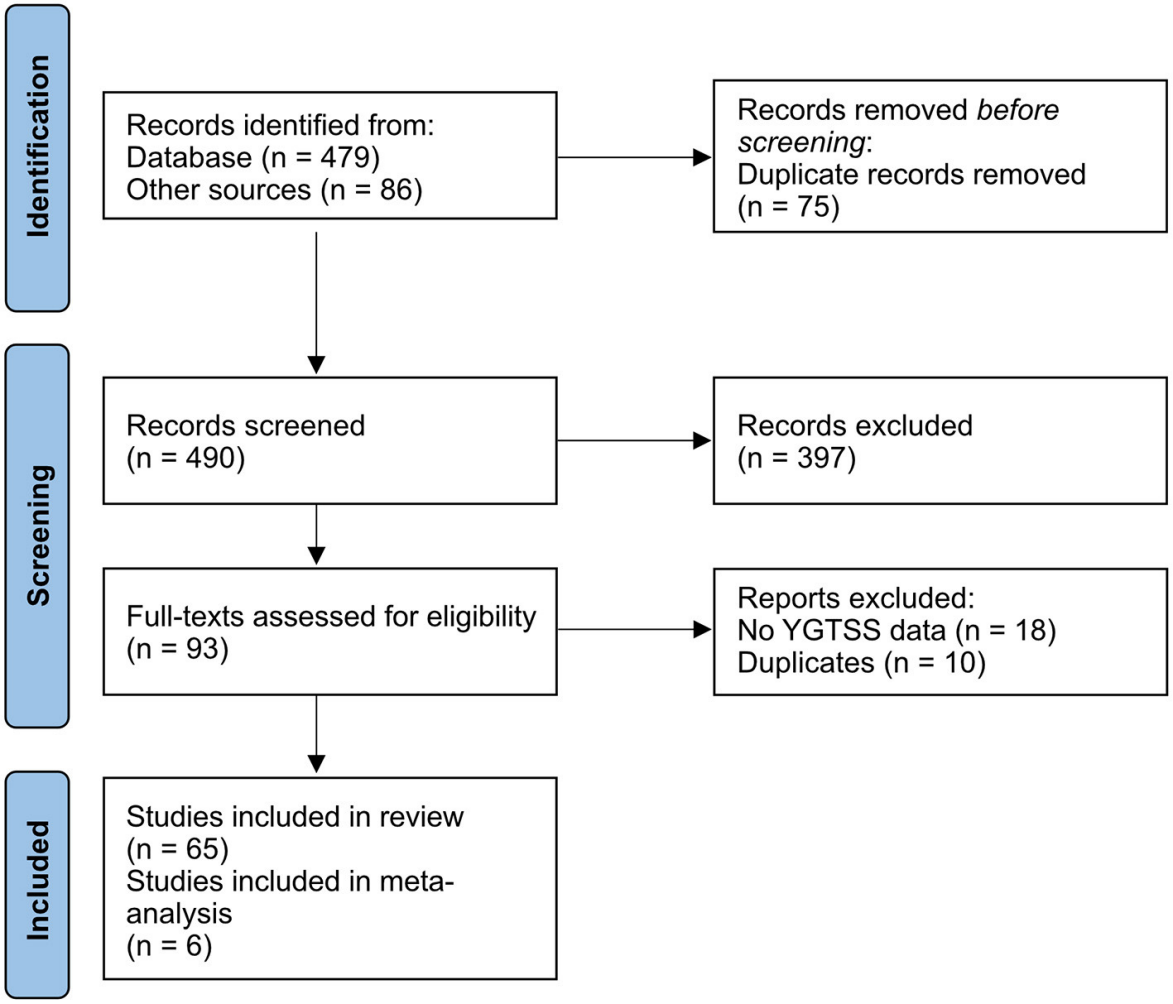
Adapted PRISMA 2020 flow diagram (44).

The PubMed search of the existing literature on the clinical outcome of DBS in TS patients identified 479 articles. In addition, the meta-analyses by Baldermann et al. (36) and Xu et al. (38) yielded 57 and 29 studies, respectively. After removing duplicates (*n* = 75), abstracts were screened for the above mentioned selection inclusion criteria, which resulted in the exclusion of 397 records. Full texts of the remaining 93 articles were subsequently checked for eligibility. Among these, 18 articles were excluded because the clinical outcome was not assessed using the YGTSS or YGTSS change was not sufficiently reported (e.g., only improvement rates without baseline values). Thereafter, additional 10 studies were excluded after a thorough analysis, because the study participants had already been reported in other articles. In total, 65 studies were included, of which 58 studies were case reports or case series with an evidence level of four (45). Seven reports were randomized, double-blinded controlled trials, with an evidence level of three. The majority of RCTs had an overall low risk of bias, except for two RCTs, which had some concerns (see details in Supplementary Figure 1). One RCT needed to be excluded because YGTSS scores were only reported for the stimulation ON setting, but not for the stimulation OFF setting. Another RCT was already excluded during the full text screening, because only percentage changes were reported without raw baseline and follow-up scores. An adapted PRISMA flow diagram is displayed in Figure 1.

### Individual Participant Data

**Figure 2:**
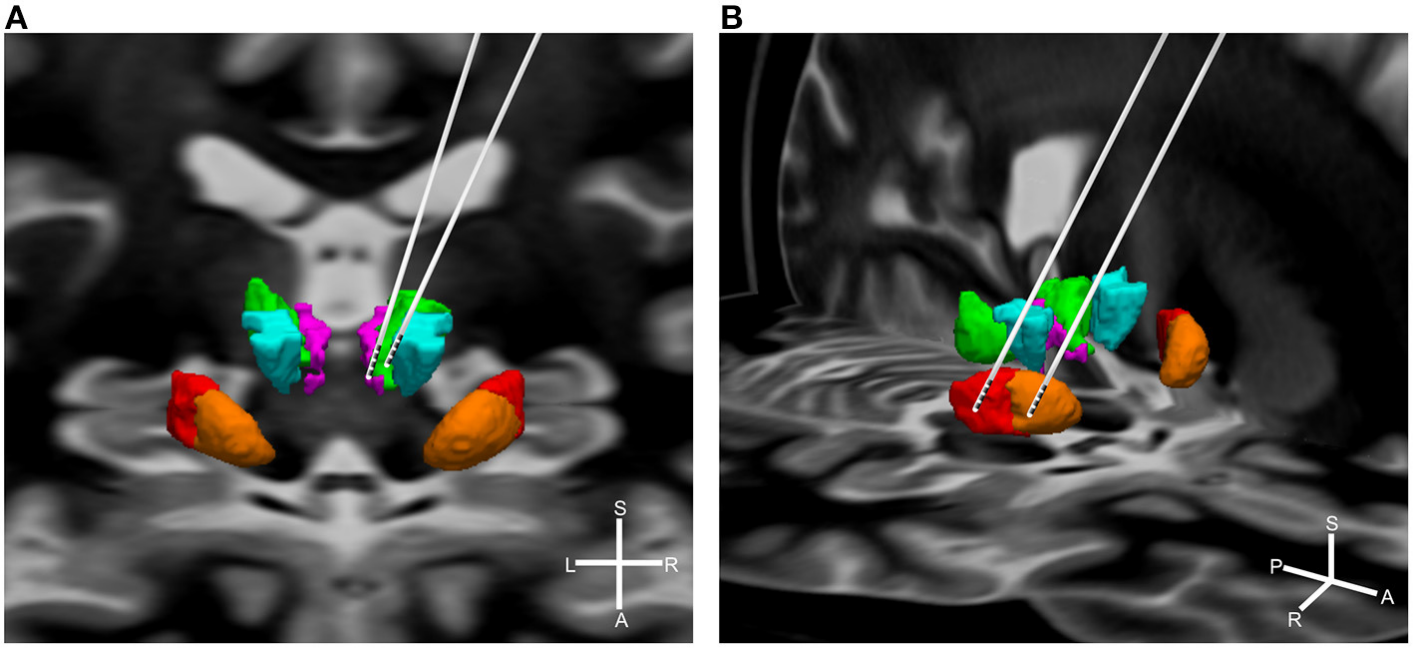
Simplified visualization of DBS electrodes of the different targets. Shown are the target regions: green = CM; purple = Pf; turquoise = Voi; red = pvlGPi; orange = amGPi. For illustration purposes targets are displayed unilateral only. **(A)** Thalamic targets: left electrode = CM-Pf; right electrode = CM-Voi. Background shows the coronal section of a brain MRI. **(B)** Pallidal targets: left electrode = pvlGPi; right electrode = amGPi. Background shows the horizontal section of a brain MRI. Graphics were generated using the DISTAL atlas (120) and MNI PD25 atlas (121). S, superior; A, anterior; L, left; R, right.

In total, 65 studies with 376 patients were included in the final analysis (see Table 1 for a detailed overview of the included studies). Most of the included patients were male (75.63%) and the median age was 30.5 years (range: 15–50 years). Of those 376 patients, 96 (25.53%) were stimulated in the CM-Voi, 59 (15.69%) in the CM-Pf, 100 (26.6%) in the amGPi, and 81 (21.54%) in the pvlGPi. The four targets are visualized in Figure 2. The ventral anterior/ventrolateral thalamus (VA/VL) was targeted in 11 patients (2.93%). In four patients, the thalamus was indicated as target, but not further specified. Similarly, in one case, the GPi without further specification was reported as the target. In two cases, both amGPi and pvlGPi were stimulated. The anterior limb of internal capsule/nucleus accumbens (ALIC/NAc) was targeted in eight patients (2.13%). In two other cases electrodes were implanted in the globus pallidus externus (GPe). A total of 12 patients received electrodes in two target areas. In two patients the thalamus and pvlGPi were targeted; however, the thalamus was not further specified. The CM-Voi and ALIC/NAc were targeted in three patients, while the CM and ALIC/NAc were targeted in one patient. Electrodes in both the amGPi and ALIC/NAc were implanted in two patients and three patients received electrodes in both the pvlGPi and the subthalamic nucleus (STN). In one patient, electrodes were implanted in the region of the ALIC and the bed of the nucleus of stria terminalis. In another two patients the fields of forel (subthalamus) were targeted. Although most patients received bilateral DBS, six patients underwent unilateral DBS in the pvlGPi and one patient in the amGPi.

**Table 1:** Overview of included studies (*n* = 65).

**References**	**Level of evidence**	***N***	**Target(s)**	**Follow-up**	**Primary outcome measure**	**Mean improvement %**
Diederich et al. (49)	4	1	pvlGPi	14 mo	YGTSS100	46.99
Bajwa et al. (50)	4	1	CM-Spv-Voi	24 mo	YGTSS50	63.64
Kuhn et al. (51)	4	1	ALIC/NAc	30 mo	YGTSS100	41.11
Maciunas et al. (52)	3	5	CM-Pf	3 mo	YGTSS100	43.60
Shahed et al. (53)	4	1	pvlGPi	12 mo	YGTSS100	73.33
Shields et al. (54)	4	1	CM	3 mo	YGTSS100	45.57
Dehning et al. (55)	4	4	pvlGPi	5–12 mo	YGTSS100	41.32
Kuhn et al. (56)	4	1	ALIC/NAc	10 mo	YGTSS100	51.85
Neuner et al. (57)	4	1	ALIC/NAc	36 mo	YGTSS100	44.00
Servello et al. (58), Servello et al. (59)*	4	6	Voi/CM-Pf (2), ALIC/NAc (1), Voi/CM-Pf + ALIC/NAc (3)	10–34 mo	YGTSS100	49.12
Burdick et al. (60)	4	1	ALIC/NAc	30 mo	YGTSS50	−14.81
Marceglia et al. (61)	4	7	Voi/CM-Pf	6–48 mo	YGTSS100	33.01
Ackermans et al. (62)	3	6	CM-Spv-Voi	12 mo	YGTSS50	47.62
Pullen et al. (63)	4	1	CM-Pf	18 mo	YGTSS100	94.81
Kaido et al. (64)	4	3	CM-Pf-Voi	12 mo	YGTSS100	36.14
Kuhn et al. (65)	4	2	VA/VL	12 mo	YGTSS100	85.98
Lee et al. (66)	4	1	CM-Pf	18 mo	YGTSS100	58.43
Martínez-Fernández et al. (67)*	4	6	amGPi (3), pvlGPi (3)	3–24 mo	YGTSS100	24.92
Rzesnitzek et al. (68)	4	1	CM-Pf	13 mo	YGTSS100	83.12
Savica et al. (69)	4	3	CM-Pf	12 mo	YGTSS100	69.73
Dong et al. (70)	4	2	pvlGPi (unilateral)	12 mo	YGTSS100	55.88
Duits et al. (71)	4	1	CM-Spv-Voi	23 mo	YGTSS50	7.14
Sachdev et al. (72)	4	1	ALIC/NAc	7 mo	YGTSS100	79.37
Massano et al. (73)	4	1	amGPi	24 mo	YGTSS100	60.49
Motlagh et al. (74)	4	8	Tha (4), pvlGPi (2), Tha + pvlGPi (2)	6–107 mo	YGTSS50	39.80
Okun et al. (75)	3	5	CM	6 mo	YGTSS100	19.43
Piedimonte et al. (76)	4	1	GPe	6 mo	YGTSS100	70.51
Dehning et al. (77)	4	6	pvlGPi	12–60 mo	YGTSS100	68.06
Dong et al. (78)	4	1	pvlGPi	39 mo	YGTSS100	92.86
Huasen et al. (79)	4	1	amGPi	12 mo	YGTSS100	55.42
Nair et al. (29)	4	4	amGPi	3–26 mo	YGTSS100	90.96
Patel and Jimenez-Shahed (80)	4	1	GPi	6 mo	YGTSS100	52.81
Pourfar et al. (81)	4	1	CM-Spv-Voi	14 mo	YGTSS100	48.86
Sachdev et al. (82), Cannon et al. (83)	4	17	amGPi (15), amGPi + ALIC/NAc (2)	4–46 mo	YGTSS100	54.21
Zhang et al. (84)	4	12	pvlGPi	13–80 mo	YGTSS100	52.13
Kefalopoulou et al. (85), Morreale et al. (86)	4	15	amGPi (12), pvlGPi (2)	6 mo	YGTSS100	50.54
Wardell et al. (87)	4	4	amGPi	14–48 mo	YGTSS100	38.66
Cury et al. (88)	4	1	CM-Pf	18 mo	YGTSS100	70.53
Huys et al. (89)	4	8	VA/VL	12 mo	YGTSS100	55.75
Smeets et al. (90)	4	5	amGPi (4), GPe (1)	12–38 mo	YGTSS50	74.23
Testini et al. (91)	4	11	CM-Pf	2–91 mo	YGTSS100	51.97
Zhang et al. (92)	4	24	pvlGPi (4 unilateral)	12 mo	YGTSS100	57.84
Akbarian-Tefaghi et al. (93)	4	15	amGPi	17–82 mo	YGTSS100	45.45
Dwarakanath et al. (94)	4	1	amGPi	9 mo	YGTSS100	72.45
Neudorfer et al. (95)	4	2	FF H1	12–18 mo	YGTSS100	76.54
Picillo et al. (96)	4	1	CM-Pf	12 mo	YGTSS100	7.69
Welter et al. (97)	3	16	amGPi	6-12 mo	YGTSS100	40.24
Azimi et al. (98)	4	6	amGPi	12 mo	YGTSS100	62.56
Doshi et al. (99)	4	2	amGPi	18 mo	YGTSS100	64.56
Dowd et al. (100)	4	12	CM-Pf-Voi	6–58 mo	YGTSS100	50.59
Kano et al. (101)	4	2	CM-Pf-Voi	29–35 mo	YGTSS100	34.13
Richieri et al. (102)	4	1	VA/VL	48 mo	YGTSS50	74.36
Brito et al. (103)	4	5	CM-Pf	12 mo	YGTSS100	30.00
Kakusa et al. (104)	4	1	CM + ALIC/NAc	12 mo	YGTSS100	84.29
Rossi et al. (105)	4	1	amGPi (unilateral)	26 mo	YGTSS100	87.10
Zhang et al. (106)	4	1	pvlGPi	3 mo	YGTSS100	53.19
Zhang et al. (107)	4	10	pvlGPi	24–96 mo	YGTSS100	81.43
Zhu et al. (108)	4	3	pvlGPi + STN	6 mo	YGTSS100	36.60
Duarte Batista et al. (109)	4	1	ALIC/BST	12 mo	YGTSS100	81.00
Servello et al. (30, 58, 110, 111), Porta et al. (112, 113), Marceglia et al. (114)	4	57	Voi-CM-Pf (41), amGPi (14), ALIC/NAc (2)	24–48 mo	YGTSS100	38.94
Andrade et al. (115), Heiden et al. (32)	4	7	CM-Voi	6 mo	YGTSS100	42.22
Kimura et al. (116)	4	25	CM-Pf	36 mo	YGTSS100	56.59
Müller-Vahl et al. (117)	3	10	CM-Voi (4), pvlGPi (6)	8–108 mo	YGTSS50	26.96
Sun et al. (118)	4	6	pvlGPi	26–48 mo	YGTSS100	59.62
Baldermann et al. (119)	4	8	CM-Voi	12 mo	YGTSS100	47.73

Duplicate studies are mentioned. An additional case was added when two targets were evaluated in one patient (*).N, Number of participants; mo, months; YGTSS100, global YGTSS score; YGTSS50, YGTSS total tic score; ALIC/NAc, Anterior limb of internal capsule/nucleus accumbens; GPe, Globus pallidus externus; STN, Subthalamic nucleus; FF H1, H1 Field of Forel; Tha, Thalamus.

### Clinical Outcomes Analysis

**Figure 3:**
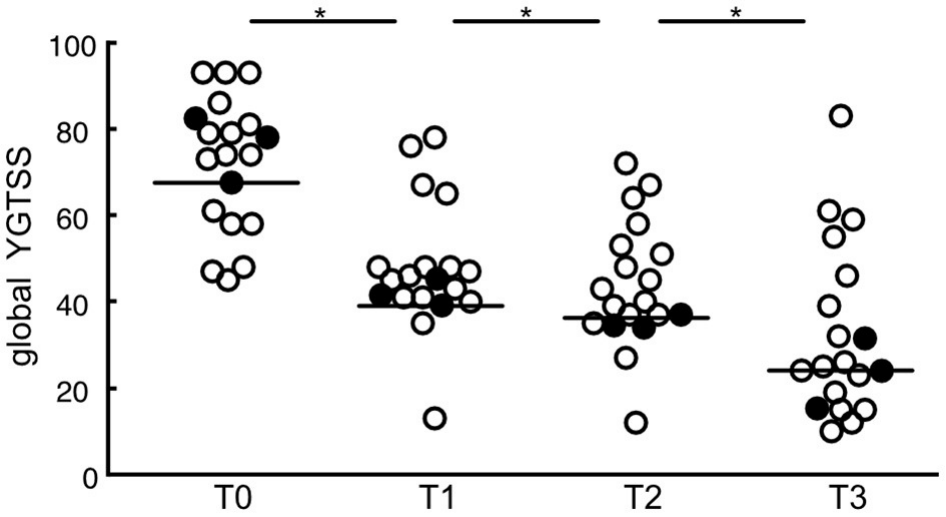
Scatterplots of global YGTSS scores for all targets combined at different postoperative time points (T0: baseline; T1: ≤ 6 months; T2: ≤ 12 months; T3: >12 months). Circles represent individual studies; color-filled circles represent more heavily weighted studies (more participants). Horizontal bars show the median values for each target. Significant differences between time points are indicated with asterisks (*p* < 0.05).

Global YGTSS scores for all targets combined were significantly reduced at maximum follow-up (*n* = 343, *Z* = −15.97, *p* < 0.001). The follow-up period ranged from 3 to 91 months (*Mdn* = 25 months). The median YGTSS score decreased from 79.92 points (*IQR* = 13.25) to a post-surgery median of 34.69 points (*IQR* = 20.93), which represents a median reduction rate of 56.59%. Also, 69.4% (*n* = 238) of the patients experienced a symptom reduction of more than 50% at maximum follow-up. Moreover, global YGTSS scores at different postoperative time points (T1: ≤ 6 months; T2: ≤ 12 months; T3: >12 months) differed significantly from postoperative baseline scores (T0). DBS resulted in a YGTSS median reduction of 34 points at T1 (*n* = 201, *Z* = −12.27, *p* < 0.001). At T2, global YGTSS scores were reduced by a median of 37 (*n* = 190, *Z* = −11.87, *p* < 0.001), whereas median scores decreased by 53.93 at T3 (*n* = 123, *Z* = −9.65, *p* < 0.001). Interestingly, clinical efficacy increased significantly over time after surgery. A Friedman's test showed a significant difference between global YGTSS scores at T0, T1, T2, and T3 [*n* = 73, χ^2^_(3)_ = 207.14, *p* < 0.001]. Dunn's *post-hoc* tests revealed that median YGTSS scores decreased from T0 to T1, from T1 to T2, and from T2 to T3 (T0: *Mdn* = 67.56, *IQR* = 10.44; T1: *Mdn* = 39.12, *IQR* = 6.18; T2: *Mdn* = 37.00, *IQR* = 2.25; T3: *Mdn* = 24.07, *IQR* = 0), which was statistically significant in all cases after Bonferroni adjustments (*p* < 0.001). YGTSS outcomes for the different time points are depicted in Figure 3.

Analysis of secondary tic-related outcome measures revealed that the median of YGTSS total tic scores decreased from 39.12 points (*IQR* = 10) to 19.0 points (*IQR* = 13) at maximum follow-up (range: 3–107 months, *Mdn* = 12 months), which equals a median symptom reduction rate of 50.43% (*n* = 159, *Z* = −10.90, *p* < 0.001). Results for the MRVRS showed a median reduction of 35.54% at maximum follow-up (Pre: *Mdn* = 14.00, *IQR* = 4.06; Post: *Mdn* = 9.00, *IQR* = 7.70, *n* = 64, *Z* = −6.57, *p* < 0.001). The follow-up period for the MRVRS ranged from 3 to 84 months (*Mdn* = 12 months). Regarding comorbid symptoms, the median of YBOCS scores decreased from 20 points (*IQR* = 10.82) to 11.45 points (*IQR* = 7.51) at maximum follow-up (range: 3–107 months, *Mdn* = 34 months), representing a median reduction rate of 43.23% (*n* = 206, *Z* = −11.84, *p* < 0.001). Of these patients, 68.4% (*n* = 141) experienced at least a 35% reduction of OCD, which is the criterion to be considered a responder (122). Finally, the BDI median score declined by a reduction of 50% from 25.70 points (*IQR* = 13.40) to 13.85 points (*IQR* = 11.30) at maximum follow-up, which ranged from 3 to 49.5 months (*Mdn* = 23.5 months). This reduction was also statistically significant (*n* = 110, *Z* = −7.71, *p* < 0.001).

### Subgroup Analysis

**Figure 4:**
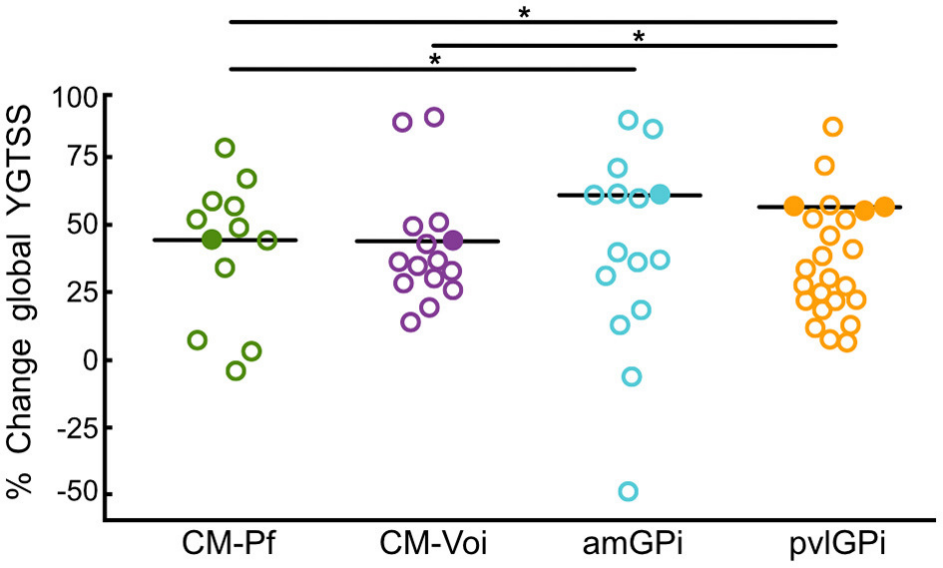
Scatterplots of global YGTSS percentage change scores for the different targets at T2 (6–12 months after DBS surgery). Circles represent individual studies; color-filled circles represent more heavily weighted studies (more participants). Horizontal bars show the median values for each target. Significant differences between targets are indicated with asterisks (*p* < 0.05).

Wilcoxon signed-rank tests revealed that stimulation of all targets resulted in a significant global YGTSS reduction after up to 12 months (see Table 2). Importantly, these target-specific YGTSS percentage changes differed significantly [*n* = 172, χ^2^_(3)_ = 21.41, *p* < 0.001]. Dunn's pairwise tests showed that the median YGTSS percentage change was significantly larger for pvlGPi compared to CM-Pf (*p* < 0.001) and CM-Voi (*p* = 0.006). Additionally, the median percentage change was significantly larger after amGPi compared to CM-Pf (*p* = 0.017). Other pairwise comparisons were not statistically significant. YGTSS outcomes for the different targets are depicted in Figure 4.

**Table 2:** Overview of global YGTSS outcomes for the different targets at T2 (6–12 months post-operatively).

**Target**	***N***	**Pre-DBS median**	**Post-DBS median**	**Median reduction**	**Median % change**	***p*-value**
CM-Pf	36	79.92 (0.00)	43.80 (0.00)	36.12 (0.00)	45.20 (0.00)	<0.001
CM-Voi	55	67.56 (0.00)	37.00 (0.00)	30.56 (0.00)	45.23 (0.00)	<0.001
amGPI	20	76.33 (8.09)	28.67 (22.67)	47.33 (23.83)	62.45 (29.36)	<0.001
pvlGPi	61	74.00 (8.40)	34.00 (3.55)	42.80 (15.50)	57.84 (13.40)	<0.001

*Measures of dispersion in brackets are interquartile ranges. P-values represents the results of Wilcoxon signed-rank tests comparing pre- and post-surgery global YGTSS scores at T2 for each target*.

**Figure 5:**
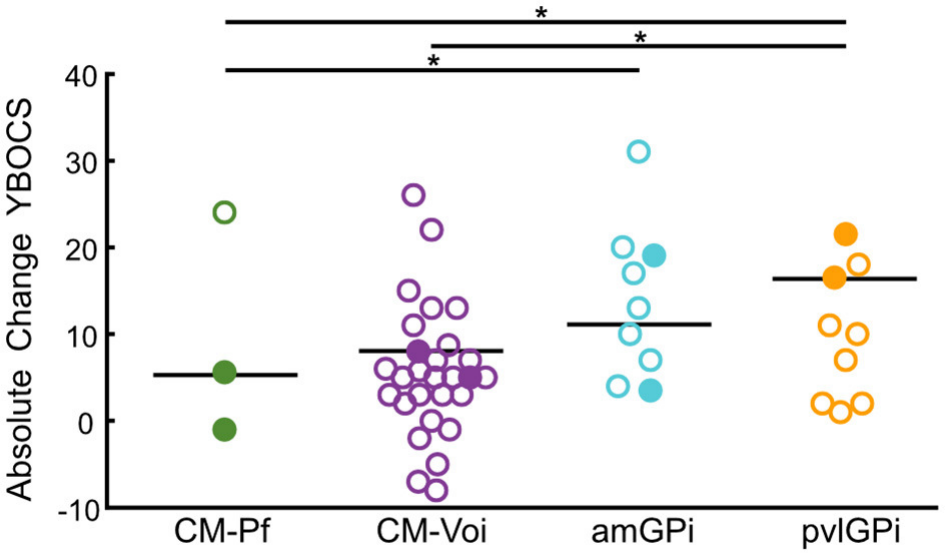
Scatterplots of YBOCS absolute change scores for the different targets at maximum follow-up. Circles represent individual studies; color-filled circles represent more heavily weighted studies (more participants). Horizontal bars show the median values for each target. Significant differences between targets are indicated with asterisks (*p* < 0.05).

Furthermore, Wilcoxon signed-rank tests showed that stimulation of the CM-Voi, amGPi, pvlGPi, but not the CM-Pf resulted in a significant reduction of YBOCS scores at maximum follow-up (range: 3–84 months, *Mdn* = 48 months) (see Table 3). Importantly, only 3 studies were included in the CM-Pf target group (*n* = 11) with a maximum follow-up period of 6 months. Subgroup analysis of the YBOCS absolute change scores showed significant differences across targets, as determined by a Kruskal-Wallis test [*n* = 143, χ^2^_(3)_ = 26.58, *p* < 0.001]. Bonferroni corrected *post-hoc* analysis indicated that the median YBOCS absolute change after pvlGPi stimulation was significantly higher than after CM-Voi DBS (*p* = 0.004) and CM-Pf DBS (*p* < 0.001). Additionally, the median absolute change was significantly greater for amGPi DBS compared to CM-Pf DBS (*p* = 0.011). Other pairwise comparisons were not statistically significant. YBOCS outcomes for the different targets are depicted in Figure 5.

**Table 3:** Overview of YBOCS outcomes for the different targets after DBS surgery at maximum follow-up.

**Target**	***N***	**Pre-DBS median**	**Post-DBS median**	**Median reduction**	**Median % change**	***p*-value**
CM-Pf	11	17.60 (5.00)	7.00 (11.60)	5.60 (6.60)	44.44 (50.13)	0.102
CM-Voi	73	20.17 (3.17)	11.45 (0.45)	8.72 (2.92)	43.23 (0.00)	<0.001
amGPI	36	19.50 (11.43)	10.69 (4.12)	11.50 (15.55)	55.17 (46.42)	<0.001
pvlGPi	23	24.70 (7.00)	3.20 (9.30)	16.50 (10.50)	87.04 (30.15)	<0.001

*Measures of dispersion in brackets are interquartile ranges. P-values represents the results of Wilcoxon signed-rank tests comparing pre- and post-surgery YBOCS scores at maximum follow-up for each target*.

### Meta-Analyses

**Figure 6:**
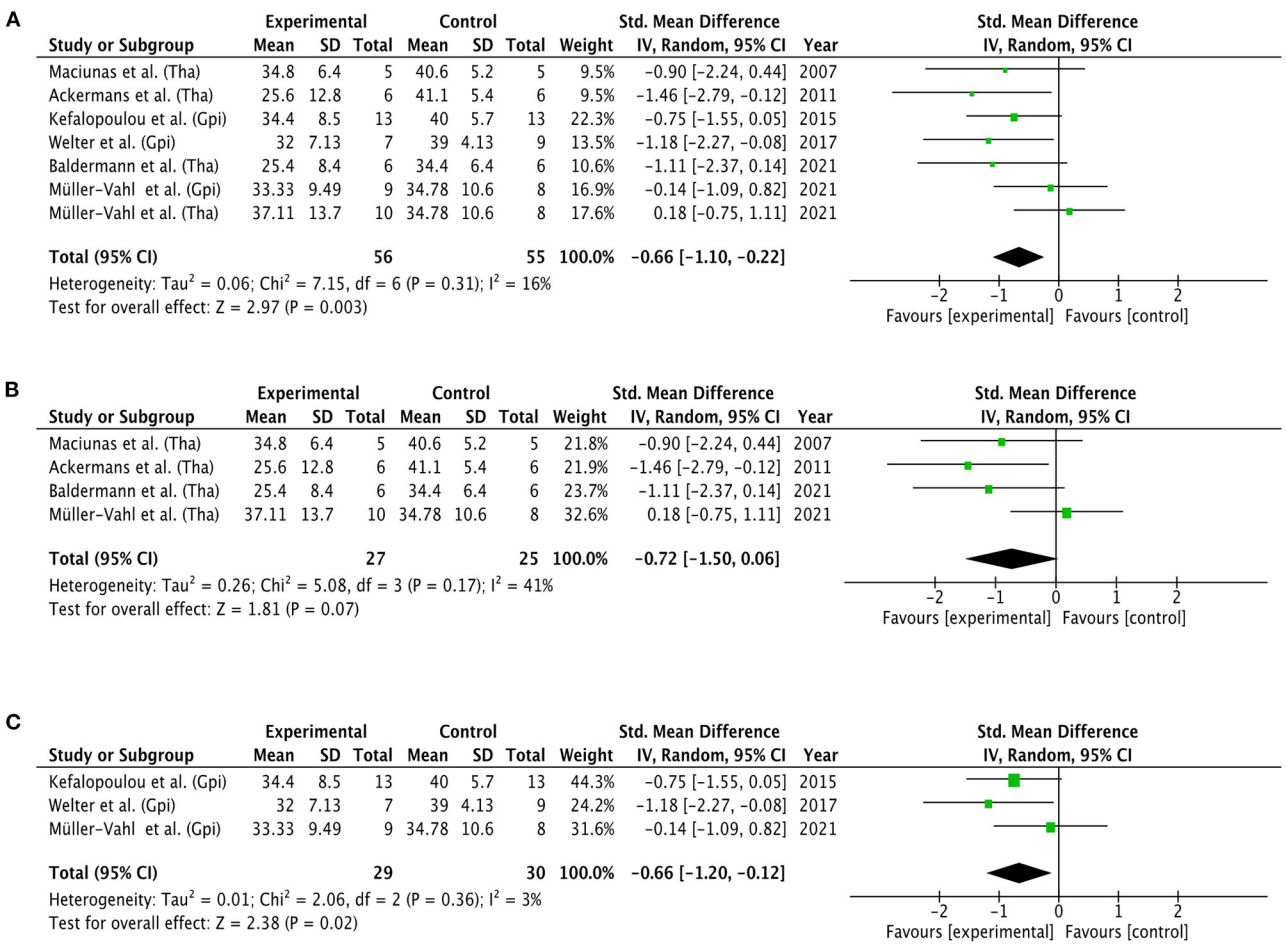
Forest plots of RCTs. Mean YGTSS total tic scores were compared between experimental conditions (DBS ON) vs. control conditions (DBS OFF). **(A)** General effect of DBS for both thalamic and pallidal targets. **(B)** Effect of DBS for thalamic targets. **(C)** Effect of DBS for pallidal targets. Targets were not further specified. Graphics were created with the Review Manager 5.4.1. (48). GPi, globus pallidus internus; Tha, thalamus; CI, confidence interval.

Three separate meta-analyses of randomized controlled and double-blinded trials were conducted with the YGTSS total tic score as primary outcome measure (see Figure 6). The first meta-analysis, which included six studies (FU range = 0.23–6 months, *Mdn* = 3 months), showed a significant overall effect of the experimental condition (DBS ON) over the control condition (DBS OFF) for thalamus and GPi targets combined. The test of heterogeneity was not significant, and the overall effect size was −0.66 (CI: −1.10, −0.22). The second meta-analysis for thalamic DBS included four studies with a total of 27 patients in the experimental group and 25 patients in the control group (FU range = 0.23–6 months, *Mdn* = 3 months). The test for the overall effect was not significant at 0.05 level (*p* = 0.07), indicating that YGTSS tic scores did not significantly differ between the experimental and control condition. The overall effect size was −0.72 (CI: −1.50, 0.06). In the contrary, results of the third meta-analysis for pallidal DBS (FU = 3 months) showed a significant overall effect of GPi DBS (*p* = 0.02), favoring stimulation ON over stimulation OFF. A non-significant heterogeneity and overall effect size of −0.66 (CI: −1.20, −0.12) were observed.

## Discussion

### Summary of Main Findings

Here, we provide an up-to-date overview of the existing literature to examine the clinical efficacy of DBS in patients with TS. Analysis of global YGTSS scores of 343 individual patients revealed that DBS of all targets combined is capable of reducing TS symptomatology. At maximum follow-up, two-thirds of patients experienced a symptom reduction of more than 50%. Considering the time course of symptom improvement after DBS-surgery, our results show that global YGTSS scores were already reduced after 6 months. Importantly, thereafter the clinical benefits of DBS increased even further. Moreover, the present results revealed that DBS resulted in significant reductions of other tic-related outcome measures (MRVRS, YGTSS total tic score) as well as comorbidities (YBOCS, BDI). The meta-analysis of six RCTs including thalamic and pallidal targets further confirmed the clinical efficacy of DBS.

Additionally, we compared the clinical outcomes of the most commonly used DBS targets, namely CM-Pf, CM-Voi, amGPi, and pvlGPi. Stimulation of all targets resulted in a significant reduction of global YGTSS scores between 6 and 12 months. However, stimulation of the GPi led to an even larger reduction rate of tic symptoms compared to thalamic stimulation. Specifically, pvlGPi DBS showed higher reduction rates of global YGTSS scores compared to CM-Pf and CM-Voi DBS. Reduction rates were also greater for amGPi DBS compared to CM-Pf DBS. Results of the two separate meta-analyses revealed a significant effect for GPi stimulation, but not for thalamic stimulation. Moreover, stimulation of all targets except for the CM-Pf resulted in a significant reduction of YBOCS scores at maximum follow-up. Also, pvlGPi DBS led to increased OCD symptom reduction compared to CM-Pf and CM-Voi DBS at maximum follow-up. Similarly, stimulation of amGPi led to increased OCD symptom reduction compared to CM-Pf stimulation.

### Interpretation of Main Findings

Based on the present results, we suggest that DBS is capable of reducing TS symptomatology in patients with treatment-refractory TS, which is in line with previous research (36, 37, 123). DBS significantly reduces tic-related symptoms as well as comorbid OCD and affective symptoms in TS patients. The latter finding is of great importance, since it is common that patients with TS exhibit at least one comorbid disorder (3, 15, 124). Moreover, time appears to play an important role in DBS for TS, as the beneficial effects of DBS seem to increase up to more than 1 year after surgery. Recent evidence implicates that this is not the case with conservative therapies, including pharmacological and behavioral therapy, which effects tend to decline over time (123). The individual optimization of stimulus parameters, especially during the first 6 months after surgery, likely contributes to this particular time course of DBS effects (74). Of note, our results are mainly based on the analysis of case reports or case series with an evidence level of four (45). The meta-analysis for all targets combined, which also pointed to the efficacy of DBS in TS, included only six RCTs with several limitations including a high heterogeneity in terms of time frame, procedure, outcome measures and target selection. In order to move away from the experimental use of DBS for TS patients, additional randomized controlled and double-blinded trials are needed. At the same time, RCTs with larger cohorts are almost impossible in TS because the number of candidates for DBS may not be sufficient. Nevertheless, future RCTs should strive to use consistent and comparable study designs.

Importantly, the present results demonstrate that stimulation of all targets lead to a significant tic reduction following DBS surgery. Similarly, stimulation of all targets except for the CM-Pf result in significant reductions of OCD symptoms. Results of the subgroup analyses also indicate that the clinical outcomes of DBS differ among the four targets. However, these results should be interpreted with great caution due to several reasons. On the one hand, we cannot rule out the possibility that the results of the subgroup analysis are influenced by our categorization of the individual targets. We have tried to categorize the targets as accurately as possible based on the description of the target locations in the original articles. However, especially in the two thalamic target groups, the individual targets within a categorization are likely to vary, because of the size as well as the complex nomenclature of the thalamus (125, 126). Also, even if authors specify the same surgical target, targets can still be slightly different. For example, personal correspondences showed that the CM-Voi target used by Servello et al. (127) is located 2 mm further anterior to the CM-Voi target of Visser-Vandewalle et al. (21, 110). Additionally, the actual volume of tissue activated (VTA) is highly dependent on factors such as the exact electrode position, stimulation settings, and individual anatomy. Furthermore, it cannot be ruled out that the results are confounded by a systematic bias in patient selection. Because of the relatively small sample sizes in target groups, clinical outcomes may be influenced by the patient selection of a single center, as patient selection processes may differ from site to site. Certain selection criteria, such as age, tic severity and impairment were shown to significantly influence clinical outcomes after DBS (36). Regarding the post-surgery time periods included in our analyses, it should be kept in mind that tic reduction rates after 6 to 12 months were compared between targets; meaning that the present analysis of the YGTSS showed differences between the targets up to 1 year after surgery. On the contrary, for the YBOCS, targets were compared at maximum follow-up, ranging from 3 to 84 months, which is a very broad time period. Similarly, studies included in the meta-analysis for thalamic DBS ranged from 7 days to 6 months, which is still a broad time period. Based on the present findings, one may argue that it is challenging to compare such temporally heterogeneous results.

Nonetheless, results of the subgroup analyses particularly emphasize the high capability of pallidal DBS to reduce tic symptoms up to 1 year following DBS surgery. In line with our findings, pvlGPi has proven to be an effective target for patients with other motor dysfunctions, such as Parkinson's disease and dystonia (33, 128–130). Therefore, the pvlGPi is also preferably chosen for DBS in TS patients with dystonic tics (67, 85). Given its anatomical connections to sensorimotor regions, the modulation of these fibers seem like a probable mechanism of action for pvlGPi DBS (28, 32). However, stimulation of projections from pvlGPi to sensorimotor networks was found to correlate negatively or not at all with tic improvement (31, 131). The amGPi was previously thought to be a particularly effective target for TS patients with comorbid OCD symptoms, but according to the present results, it may also play an equally important role in tic reduction (30). In line with this, registry data demonstrated that amGPi DBS resulted in the greatest tic improvement after 1 year compared to CM, pvlGPi and ALIC DBS; however differences between targets were not significant (37). Concurrently, connectivity from the amGPi to limbic and associative networks positively correlated with tic improvement (31, 131). Interestingly, activation of the sensorimotor pallido-subthalamic pathway was more predictive of OCD symptom improvement compared to the associative pallido-subthalamic pathway (131). This agrees with our findings, which demonstrated the high capability of pvlGPi and amGPi DBS in reducing OCD symptoms. Surprisingly, the current findings partly differ from what we know from previous reports and are not entirely consistent with the functionally distinction of sensorimotor, associative, and limbic pathways. It should be noted that TS is no pure motor disorder (132). Heterogeneity and complexity of the disorder might partly explain the tic improvement following amGPi DBS and OCD symptom improvement after pvlGPi DBS (131, 133). Additionally, the different targets might improve TS symptomatology through different functional mechanisms, such as direct inhibition of tic execution or enhancement of the ability to suppress tics (134, 135). However, the exact causal relationships are not understood, and further research is needed to explain this inverse differentiation of the pallidal DBS targets.

Beyond that, the present results suggest that thalamic DBS yields lower tic reduction rates compared to pallidal DBS up to 12 months postoperatively. To our knowledge, no significant differences have to date been found between targets in terms of tic reduction rates (36–38, 136). Only a few studies compared the clinical effects of thalamic stimulation with those of pallidal stimulation, which indeed pointed to a superior effect of the latter, but only up to 3 months (117, 137, 138). However, as our findings show, it may take at least 1 year for the positive effects of DBS to fully develop. Accordingly, YGTSS reduction was shown to be greater at least 1 year after CM-Pf DBS compared to <1 year (91). Moreover, although the initial positive effects of GPi DBS have been shown to decrease several years after surgery, the beneficial effects from CM-Voi DBS were ongoing in a subset of patients (117, 139). Based on this, we cannot rule out differences in clinical time courses between targets, but long-term results are rare and further investigations are needed. Apart from that, results of our meta-analysis revealed a non-significant effect of DBS for thalamic targets. It should be noted that this finding was predominantly shaped by a single RCT favoring stimulation OFF over stimulation ON, which was weighted with 32.6% (for details see Figure 6). According to the authors, results of this trials might be influenced by poor compliance, placebo effect, and high infection rate (117). Also, in three patients, electrode positions did not correspond to the planned target point and extended into subthalamic regions, which in turn may have compromised optimal stimulation settings, eventually resulting in under-stimulation (117). Furthermore, our results revealed that thalamic DBS targets are less capable of alleviating OCD symptoms than pallidal targets. In particular, CM-Pf DBS was found to have no effect at all. This result is rather surprising, because of the connections between the CM-Pf and limbic regions, especially the nucleus accumbens (23–25). However, it should be noted that only 3 studies were included in the CM-Pf target group (*n* = 11) with a maximum follow-up period of 6 months. As already discussed above, the results are also highly dependent on the patient selection and the type and severity of the OCD symptoms. Centers tend to target the amGPi or ALIC/NAc for patients with more severe OCD symptoms, while CM-Pf is preferably chosen for patients with predominant tic symptoms (140).

Finally, it needs to be mentioned that despite the effectiveness of the various DBS targets, other factors also play a role in the selection of targets. In the present review, no differences in side effects between the targets have been taken into account, because a quantitative evaluation of adverse events was not feasible due to lack of information. Ideally the safety of DBS should also be assessed in the same way. Side effects may vary across the four targets, which could influence the final decision on target selection for DBS of individual patients. Some other technical details are also not considered, such as the substantial amount of total energy needed for GPi stimulation compared to thalamic stimulation, which may result in reduced battery life duration, leading to more frequent battery replacements in the case of non-rechargeable implanted pulse generators (141).

To sum up, it should be emphasized that the present results do not provide an answer to the question of which target is more clinically relevant for the treatment of TS. Rather, they highlight the importance of considering which target might be the best choice for the individual patients based on specific symptoms and individual characteristics. Future studies might focus on defining precise criteria and guidelines for the target selection for DBS in TS.

### Future Directions for DBS Targeting in TS

Connectomic DBS represents a unique opportunity to guide target selection in psychiatric disorders that are heterogenous, such as Tourette Syndrome (39, 142, 143). The application of DTI tractography has the great potential to shift the focus away from identifying one appropriate target for TS and instead enable for personalized and symptom-specific targeting. Specifically, a connectomic approach may allow to display the fiber pathways associated with specific symptom improvement. Identification of such connectivity patterns could potentially lead to the optimization of targets or discovery of new targets. Several studies have investigated structural connectivity patterns in DBS for TS (31, 32, 103, 115, 131, 144). Importantly, studies showed that the VTA of the target alone did not predict the clinical efficacy of DBS for TS (103, 145). Instead, results of several studies indicated that the connectivity between the VTA and cortical regions was linked to the clinical outcome after DBS in TS patients (31, 32, 103, 115). However, the various targets used for DBS in TS show different connectivity profiles, and cortical networks linked to clinical improvement have been shown to differ across targets (31, 32). In particular, networks positively correlated with tic improvement included limbic and associative regions for the GPi, and sensorimotor as well as parietal-temporal-occipital regions for the thalamus. For both targets, connectivity to the cerebellum also correlated positively with tic improvement (31). This suggests that stimulation of the different targets does not result in the modulation of a single network. Rather, stimulation of the different targets might result in the modulation of distinct, maybe partly overlapping networks, which then lead to the improvement of specific symptoms via a certain functional mechanism. DBS should aim to target those symptom-specific networks, thereby allowing to treat the entire complex spectrum of TS symptoms. Further studies examining the clinical outcomes of DBS in TS with known targets using structural imaging techniques are needed to improve our understanding of the underlying DBS mechanisms and to increase the efficacy of target selection. Particularly, there is a need for studies that identify fiber pathways associated with improvement of various TS symptoms, including simple tics, complex tics, the premonitory urge, comorbid symptoms, as well as tic suppression. In addition, the functional mechanisms by which modulation of the network ultimately improves tic symptoms (e.g., by directly inhibiting tic execution or by improving the ability to suppress tics) should also be investigated.

### Limitations

There are several limitations of the present review. As already mentioned above, the most obvious limitation is that our results are mainly based on case reports and case series with a high risk of bias. In addition, not all individual data were available, and aggregate data had to be extracted for some studies. This was mitigated by weighting the data by sample size for statistical analysis. Regarding the subgroup analyses, the numbers of patients in each target group varied. Notably, the number of patients in the CM-Pf target group for the YBOCS subgroup analysis was very low. The meta-analysis for all targets combined included only six RCTs, with a high heterogeneity in terms of time frame, procedure, outcome measures and target selection. Considering that the effects of DBS continue to manifest up to more than 12 months after surgery, one could argue that the included RCTs are also generally too short. Next, when drawing conclusions, one should be aware that the included articles in the present systematic review represent a very heterogenous data pool. The significant effects might be influenced by other factors, such as patient selection, tic severity before surgery, age, sex, poor compliance, medication, placebo effect in open-label settings, or stimulation parameters. Moreover, the wide time range of the maximum follow-up is another limitation, that may influence the results systematically. Taking into account the increase in the effectiveness of DBS over time, it may be considered problematic to report aggregated follow-up scores that span more than 6 months. For global YGTSS scores, we were unable to further narrow down the time category T3 (>12 months), because of insufficient data. Therefore, no statistical analyses were reasonably possible to examine whether the beneficial effects ceased over time. For the future, the use of international registries might contribute as part of the solution for this problem (146). It would also have been worthwhile to examine whether the increase of clinical efficacy of DBS differs between the four targets. Unfortunately, this was also not possible due to insufficient data. Another limitation refers to the assessment of TS symptomatology. The diversity of symptoms is not reflected in mean scores, such as the global YGTSS or YBOCS score. Thus, the heterogeneity of tics and comorbid symptoms was not considered in the present analysis. Moreover, to evaluate the effect of DBS on more of the heterogenous symptoms of TS, it would have been helpful to include additional psychiatric scales in the final analysis, including assessments of the premonitory urge (Premonitory Urge for Tic Scale–PUTS), and quality of life (Gilles de la Tourette Syndrome-Quality of Life Scale–GTS-QoL) (147, 148). However, these assessments were very rarely used in the included studies. Lastly, no side effects of DBS were reviewed in the present work. These limitations should be considered when planning and conducting future research, especially randomized controlled and double-blinded trials.

## Conclusion

We conclude that DBS is a clinically effective treatment option for patients with treatment-refractory TS, with all targets showing comparable significant improvement rates. However, the present results suggest that reduction rates in tic symptoms may differ across targets up to 12 months after surgery. Importantly, it may take at least 1 year for the positive effects of DBS to fully develop, and therefore no conclusions can be drawn about potential differences in long-term clinical outcomes between targets. Future research might shift its focus away from identifying one appropriate target for DBS in TS and instead enable personalized and symptom-specific target selection. A first step in this direction might be the characterization of target- and symptom-specific networks modulated by DBS.

## Data Availability Statement

The original contributions presented in the study are included in the article/Supplementary Materials, further inquiries can be directed to the corresponding author.

## Author Contributions

The study has been designed by LW, TS, VV-V, JB, and PA. The literature search was conducted and data have been extracted by LW and JK. Data have been analyzed and interpreted by LW, TS, JB, and PA. The manuscript has been drafted by LW. Figures have been created by LW and PH. All authors revised and edited the manuscript.

## Funding

This study was supported by the German Research Foundation (SFB 1451, Project ID 431549029-C07) and the Marga und Walter Boll Stiftung (210-06-16).

## Conflict of Interest

The authors declare that the research was conducted in the absence of any commercial or financial relationships that could be construed as a potential conflict of interest.

## Publisher's Note

All claims expressed in this article are solely those of the authors and do not necessarily represent those of their affiliated organizations, or those of the publisher, the editors and the reviewers. Any product that may be evaluated in this article, or claim that may be made by its manufacturer, is not guaranteed or endorsed by the publisher.

## Supplementary Material

Supplementary Figure 1 Summary table of risk of bias domains in each RCT created with the risk of bias visualization (robvis) tool (149).

Supplementary Material 1 Search terms.

## References

[1] Efron D Dale RC. Tics and Tourette syndrome. J Paediatr Child Health. (2018) 54:1148–53. 10.1111/jpc.14165 30294996

[2] Leckman JF Zhang H Vitale A Lahnin F Lynch K Bondi C . Course of tic severity in Tourette syndrome: the first two decades. Pediatrics. (1998) 102:14–9. 10.1542/peds.102.1.14 9651407

[3] Eapen V Cavanna AE Robertson MM. Comorbidities, social impact, and quality of life in Tourette syndrome. Front Psychiatry. (2016) 7:97. 10.3389/fpsyt.2016.00097 27375503 PMC4893483

[4] Zinner SH Conelea CA Glew GM Woods DW Budman CL. Peer victimization in youth with Tourette syndrome and other chronic tic disorders. Child Psychiatry Hum Dev. (2012) 43:124–36. 10.1007/s10578-011-0249-y 21879319

[5] Ganos C Roessner V Münchau A. The functional anatomy of Gilles de la Tourette syndrome. Neurosci Biobehav Rev. (2013) 37:1050–62. 10.1016/j.neubiorev.2012.11.004 23237884

[6] Albin RL Mink JW. Recent advances in Tourette syndrome research. Trends Neurosci. (2006) 29:175–82. 10.1016/j.tins.2006.01.001 16430974

[7] Mink JW. The basal ganglia and involuntary movements: impaired inhibition of competing motor patterns. Arch Neurol. (2003) 60:1365–8. 10.1001/archneur.60.10.1365 14568805

[8] Leckman JF Vaccarino FM Kalanithi PSA Rothenberger A. Annotation: Tourette syndrome: a relentless drumbeat – driven by misguided brain oscillations. J Child Psychol Psychiatry. (2006) 47:537–50. 10.1111/j.1469-7610.2006.01620.x 16712630

[9] Rae CL Critchley HD Seth AK. A bayesian account of the sensory-motor interactions underlying symptoms of Tourette syndrome. Front Psychiatry. (2019) 10:29. 10.3389/fpsyt.2019.00029 30890965 PMC6412155

[10] Wang Z Maia TV Marsh R Colibazzi T Gerber A Peterson BS. The neural circuits that generate tics in Tourette's syndrome. Am J Psychiatry. (2011) 168:1326–37. 10.1176/appi.ajp.2011.09111692 21955933 PMC4246702

[11] Worbe Y Malherbe C Hartmann A Pélégrini-Issac M Messé A Vidailhet M . Functional immaturity of cortico-basal ganglia networks in Gilles de la Tourette syndrome. Brain. (2012) 135:1937–46. 10.1093/brain/aws056 22434213

[12] Polyanska L Critchley HD Rae CL. Centrality of prefrontal and motor preparation cortices to Tourette syndrome revealed by meta-analysis of task-based neuroimaging studies. NeuroImage Clin. (2017) 16:257–67. 10.1016/j.nicl.2017.08.004 28831377 PMC5554925

[13] Robertson MM. Diagnosing Tourette syndrome: is it a common disorder? J Psychosom Res. (2003) 55:3–6. 10.1016/S0022-3999(02)00580-9 12842225

[14] Leckman JF Cohen DJ. Tourette's Syndrome-tics, Obsessions, Compulsions: Developmental Psychopathology and Clinical Care. Hoboken, NJ: John Wiley & Sons Inc (1999).

[15] Gill CE Kompoliti K. Clinical features of Tourette syndrome. J Child Neurol. (2020) 35:166–74. 10.1177/0883073819877335 31608744

[16] Azrin NH Nunn RG. Habit-reversal: a method of eliminating nervous habits and tics. Behav Res Ther. (1973) 11:619–28. 10.1016/0005-7967(73)90119-8 4777653

[17] Frank M Cavanna AE. Behavioural treatments for Tourette syndrome: an evidence-based review. Behav Neurol. (2013) 27:105–17. 10.1155/2013/134863 23187152 PMC5215725

[18] Wilhelm S Peterson AL Piacentini J Woods DW Deckersbach T Sukhodolsky DG . Randomized trial of behavior therapy for adults with Tourette syndrome. Arch Gen Psychiatry. (2012) 69:795–803. 10.1001/archgenpsychiatry.2011.1528 22868933 PMC3772729

[19] Huys D Hardenacke K Poppe P Bartsch C Baskin B Kuhn J. Update on the Role of Antipsychotics in the Treatment of Tourette Syndrome. Neuropsychiatr Dis Treat. (2012) 8:95–104. 10.2147/NDT.S12990 22442630 PMC3307661

[20] Johnson MD Miocinovic S McIntyre CC Vitek JL. Mechanisms and targets of deep brain stimulation in movement disorders. Neurotherapeutics. (2008) 5:294–308. 10.1016/j.nurt.2008.01.010 18394571 PMC2517242

[21] Vandewalle V van der Linden C Groenewegen HJ Caemaert J. Stereotactic treatment of Gilles de la Tourette syndrome by high frequency stimulation of thalamus. Lancet. (1999) 353:724. 10.1016/S0140-6736(98)05964-9 10073521

[22] Hassler R Dieckmann G. Stereotaxic treatment of tics and inarticulate cries or coprolalia considered as motor obsessional phenomena in Gilles de la Tourette's disease. Rev Neurol. (1970) 123:89–100. 4932913

[23] Ilyas A Pizarro D Romeo AK Riley KO Pati S. The centromedian nucleus: anatomy, physiology, and clinical implications. J Clin Neurosci. (2019) 63:1–7. 10.1016/j.jocn.2019.01.050 30827880

[24] Sadikot AF Rymar VV. The primate centromedian–parafascicular complex: anatomical organization with a note on neuromodulation. Brain Res Bull. (2009) 78:122–30. 10.1016/j.brainresbull.2008.09.016 18957319

[25] Van der Werf YD Witter MP Groenewegen HJ. The intralaminar and midline nuclei of the thalamus. Anatomical and functional evidence for participation in processes of arousal and awareness. Brain Res Rev. (2002) 39:107–40. 10.1016/S0165-0173(02)00181-9 12423763

[26] Cacciola A Milardi D Bertino S Basile GA Calamuneri A Chillemi G . Structural connectivity-based topography of the human globus pallidus: implications for therapeutic targeting in movement disorders. Mov Disord. (2019) 34:987–96. 10.1002/mds.27712 31077436

[27] Nambu A. Somatotopic organization of the primate basal ganglia. Front Neuroanat. (2011) 5:26. 10.3389/fnana.2011.00026 21541304 PMC3082737

[28] Worbe Y Marrakchi-Kacem L Lecomte S Valabregue R Poupon F Guevara P . Altered structural connectivity of cortico-striato-pallido-thalamic networks in Gilles de la Tourette syndrome. Brain. (2014) 138:472–82. 10.1093/brain/awu311 25392196 PMC4306818

[29] Nair G Evans A Bear RE Velakoulis D Bittar RG. The anteromedial GPi as a new target for deep brain stimulation in obsessive compulsive disorder. J Clin Neurosci. (2014) 21:815–21. 10.1016/j.jocn.2013.10.003 24524950

[30] Servello D Galbiati TF Balestrino R Iess G Zekaj E Michele S . Deep brain stimulation for Gilles de la Tourette syndrome: toward limbic targets. Brain Sci. (2020) 10:301. 10.3390/brainsci10050301 32429219 PMC7287742

[31] Johnson KA Duffley G Anderson DN Ostrem JL Welter ML Baldermann JC . Structural connectivity predicts clinical outcomes of deep brain stimulation for Tourette syndrome. Brain. (2020) 143:2607–23. 10.1093/brain/awaa188 32653920 PMC7447520

[32] Heiden P Hoevels M Bayram D Baldermann JC Schuller T Huys D . Connectivity patterns of deep brain stimulation targets in patients with Gilles de la Tourette syndrome. Brain Sci. (2021) 11:87. 10.3390/brainsci11010087 33440771 PMC7826809

[33] Ackermans L Temel Y Cath D van der Linden C Bruggeman R Kleijer M . Deep brain stimulation in Tourette's syndrome: two targets? Mov Disord. (2006) 21:709–13. 10.1002/mds.20816 16463374

[34] Porta M Saleh C Zekaj E Zanaboni Dina C Bona AR Servello D. Why so many deep brain stimulation targets in Tourette's syndrome? Toward a broadening of the definition of the syndrome. J Neural Transm. (2016) 123:785–90. 10.1007/s00702-015-1494-1 26739445

[35] Viswanathan A Jimenez-Shahed J Baizabal Carvallo JF Jankovic J. Deep brain stimulation for Tourette syndrome: target selection. Stereotact Funct Neurosurg. (2012) 90:213–24. 10.1159/000337776 22699684

[36] Baldermann JC Schüller T Huys D Becker I Timmermann L Jessen F . Deep brain stimulation for Tourette-syndrome: a systematic review and meta-analysis. Brain Stimul. (2016) 9:296–304. 10.1016/j.brs.2015.11.005 26827109

[37] Martinez-Ramirez D Jimenez-Shahed J Leckman JF Porta M Servello D Meng FG . Efficacy and safety of deep brain stimulation in Tourette syndrome: the international Tourette syndrome deep brain stimulation public database and registry. JAMA Neurol. (2018) 75:353–9. 10.1001/jamaneurol.2017.4317 29340590 PMC5885852

[38] Xu W Zhang C Deeb W Patel B Wu Y Voon V . Deep brain stimulation for Tourette's syndrome. Transl Neurodegener. (2020) 9:4. 10.1186/s40035-020-0183-7 31956406 PMC6956485

[39] Horn A. The impact of modern-day neuroimaging on the field of deep brain stimulation. Curr Opin Neurol. (2019) 32:511–20. 10.1097/WCO.0000000000000679 30844863

[40] Ashkan K Rogers P Bergman H Ughratdar I. Insights into the mechanisms of deep brain stimulation. Nat Rev Neurol. (2017) 13:548–54. 10.1038/nrneurol.2017.105 28752857

[41] McIntyre CC Hahn PJ. Network perspectives on the mechanisms of deep brain stimulation. Neurobiol Dis. (2010) 38:329–37. 10.1016/j.nbd.2009.09.022 19804831 PMC2862840

[42] Deeb W Malaty I. Deep brain stimulation for Tourette syndrome: potential role in the pediatric population. J Child Neurol. (2020) 35:155–65. 10.1177/0883073819872620 31526168

[43] Porta M Servello D Sassi M Brambilla A Defendi S Priori A . Issues related to deep brain stimulation for treatment-refractory Tourette's syndrome. Eur Neurol. (2009) 62:264–73. 10.1159/000235595 19690419

[44] Page MJ McKenzie JE Bossuyt PM Boutron I Hoffmann TC Mulrow CD . The PRISMA 2020 statement: an updated guideline for reporting systematic reviews. BMJ. (2021) 372:n71. 10.1136/bmj.n71 33782057 PMC8005924

[45] French J Gronseth G. Invited article: lost in a jungle of evidence: we need a compass. Neurology. (2008) 71:1634–8. 10.1212/01.wnl.0000336533.19610.1b 19001254

[46] Higgins JP Thomas J Chandler J Cumpston M Li T Page MJ . Cochrane Handbook for Systematic Reviews of Interventions: Chichester, UK: John Wiley & Sons (2019). 10.1002/9781119536604 PMC10284251 31643080

[47] IBM Corp. IBM SPSS Statistics for Windows, Version 27.0. Armonk, NY: IBM Corp (202).

[48] The Cochrane Collaboration. Review Manager (RevMan) [Computer program]. Version 5.4. The Cochrane Collaboration (2020).

[49] Diederich NJ Kalteis K Stamenkovic M Pieri V Alesch F. Efficient internal pallidal stimulation in Gilles de la Tourette syndrome: a case report. Mov Disord. (2005) 20:1496–9. 10.1002/mds.20551 16037913

[50] Bajwa RJ de Lotbiniere AJ King RA Jabbari B Quatrano S Kunze K . Deep brain stimulation in Tourette's syndrome. Mov Disord. (2007) 22:1346–50. 10.1002/mds.21398 17580320

[51] Kuhn J Lenartz D Mai JK Huff W Lee SH Koulousakis A . Deep brain stimulation of the nucleus accumbens and the internal capsule in therapeutically refractory Tourette-syndrome. J Neurol. (2007) 254:963–5. 10.1007/s00415-006-0404-8 17410328

[52] Maciunas RJ Maddux BN Riley DE Whitney CM Schoenberg MR Ogrocki PJ . Prospective randomized double-blind trial of bilateral thalamic deep brain stimulation in adults with Tourette syndrome. J Neurosurg. (2007) 107:1004–14. 10.3171/JNS-07/11/1004 17977274

[53] Shahed J Poysky J Kenney C Simpson R Jankovic J. GPi deep brain stimulation for Tourette syndrome improves tics and psychiatric comorbidities. Neurology. (2007) 68:159–60. 10.1212/01.wnl.0000250354.81556.90 17210901

[54] Shields DC Cheng ML Flaherty AW Gale JT Eskandar EN. Microelectrode-guided deep brain stimulation for Tourette syndrome: within-subject comparison of different stimulation sites. Stereotact Funct Neurosurg. (2008) 86:87–91. 10.1159/000112429 18073521

[55] Dehning S Mehrkens JH Müller N Bötzel K. Therapy-refractory Tourette syndrome: beneficial outcome with globus pallidus internus deep brain stimulation. Mov Disord. (2008) 23:1300–2. 10.1002/mds.21930 18528896

[56] Kuhn J Lenartz D Huff W Mai JK Koulousakis A Maarouf M . Transient manic-like episode following bilateral deep brain stimulation of the nucleus accumbens and the internal capsule in a patient with Tourette syndrome. Neuromodulation. (2008) 11:128–31. 10.1111/j.1525-1403.2008.00154.x 22151046

[57] Neuner I Podoll K Lenartz D Sturm V Schneider F. Deep brain stimulation in the nucleus accumbens for intractable Tourette's syndrome: follow-up report of 36 months. Biol Psychiatry. (2009) 65:e5–6. 10.1016/j.biopsych.2008.09.030 19006786

[58] Servello D Sassi M Brambilla A Defendi S Porta M. Long-term, post-deep brain stimulation management of a series of 36 patients affected with refractory Gilles de la Tourette syndrome. Neuromodulation. (2010) 13:187–94. 10.1111/j.1525-1403.2009.00253.x 21992831

[59] Servello D Sassi M Brambilla A Porta M Haq I Foote KD . De novo and rescue DBS leads for refractory Tourette syndrome patients with severe comorbid OCD: a multiple case report. J Neurol. (2009) 256:1533–9. 10.1007/s00415-009-5159-6 19437063

[60] Burdick A Foote KD Goodman W Ward HE Ricciuti N Murphy T . Lack of benefit of accumbens/capsular deep brain stimulation in a patient with both tics and obsessive-compulsive disorder. Neurocase. (2010) 16:321–30. 10.1080/13554790903560422 20178034

[61] Marceglia S Servello D Foffani G Porta M Sassi M Mrakic-Sposta S . Thalamic single-unit and local field potential activity in Tourette syndrome. Mov Disord. (2010) 25:300–8. 10.1002/mds.22982 20108375

[62] Ackermans L Duits A van der Linden C Tijssen M Schruers K Temel Y . Double-blind clinical trial of thalamic stimulation in patients with Tourette syndrome. Brain. (2011) 134:832–44. 10.1093/brain/awq380 21354977

[63] Pullen SJ Wall CA Lee KH Stead SM Klassen BT Brown TM. Neuropsychiatric outcome of an adolescent who received deep brain stimulation for Tourette's syndrome. Case Rep Neurol Med. (2011) 2011:209467. 10.1155/2011/209467 22937332 PMC3420565

[64] Kaido T Otsuki T Kaneko Y Takahashi A Omori M Okamoto T. Deep brain stimulation for Tourette syndrome: a prospective pilot study in Japan. Neuromodulation. (2011) 14:123–8. 10.1111/j.1525-1403.2010.00324.x 21992198

[65] Kuhn J Bartsch C Lenartz D Huys D Daumann J Woopen C . Clinical effectiveness of unilateral deep brain stimulation in Tourette syndrome. Transl Psychiatry. (2011) 1:e52. 10.1038/tp.2011.51 22833207 PMC3309474

[66] Lee MW Au-Yeung MM Hung KN Wong CK. Deep brain stimulation in a Chinese Tourette's syndrome patient. Hong Kong Med J. (2011) 17:147–50. 21471596

[67] Martínez-Fernández R Zrinzo L Aviles-Olmos I Hariz M Martinez-Torres I Joyce E . Deep brain stimulation for Gilles de la Tourette syndrome: a case series targeting subregions of the globus pallidus internus. Mov Disord. (2011) 26:1922–30. 10.1002/mds.23734 21538528

[68] Rzesnitzek L Wachter T Kruger R Gharabaghi A Plewnia C. Suppression of extrapyramidal side effects of doxepin by thalamic deep brain stimulation for Tourette syndrome. Neurology. (2011) 77:1708–9. 10.1212/WNL.0b013e318236485f 22042798

[69] Savica R Stead M Mack KJ Lee KH Klassen BT. Deep brain stimulation in Tourette syndrome: a description of 3 patients with excellent outcome. Mayo Clin Proc. (2012) 87:59–62. 10.1016/j.mayocp.2011.08.005 22212969 PMC3538384

[70] Dong S Zhuang P Zhang XH Li JY Li YJ. Unilateral deep brain stimulation of the right globus pallidus internus in patients with Tourette's syndrome: two cases with outcomes after 1 year and a brief review of the literature. J Int Med Res. (2012) 40:2021–8. 10.1177/030006051204000545 23206487

[71] Duits A Ackermans L Cath D Visser-Vandewalle V. Unfavourable outcome of deep brain stimulation in a Tourette patient with severe comorbidity. Eur Child Adolesc Psychiatry. (2012) 21:529–31. 10.1007/s00787-012-0285-6 22622600 PMC3432784

[72] Sachdev PS Cannon E Coyne TJ Silburn P. Bilateral deep brain stimulation of the nucleus accumbens for comorbid obsessive compulsive disorder and Tourette's syndrome. BMJ Case Rep. (2012) 2012. 10.1136/bcr-2012-006579 22977057 PMC4544584

[73] Massano J Sousa C Foltynie T Zrinzo L Hariz M Vaz R. Successful pallidal deep brain stimulation in 15-year-old with Tourette syndrome: 2-year follow-up. J Neurol. (2013) 260:2417–9. 10.1007/s00415-013-7049-1 23884714

[74] Motlagh MG Smith ME Landeros-Weisenberger A Kobets AJ King RA Miravite J . Lessons learned from open-label deep brain stimulation for Tourette syndrome: eight cases over 7 years. Tremor Other Hyperkinet Mov. (2013) 3:tre-03-170-4428-1. 10.5334/tohm.126 24255802 PMC3822402

[75] Okun MS Foote KD Wu SS Ward HE Bowers D Rodriguez RL . A trial of scheduled deep brain stimulation for Tourette syndrome: moving away from continuous deep brain stimulation paradigms. JAMA Neurol. (2013) 70:85–94. 10.1001/jamaneurol.2013.580 23044532

[76] Piedimonte F Andreani JC Piedimonte L Graff P Bacaro V Micheli F . Behavioral and motor improvement after deep brain stimulation of the globus pallidus externus in a case of Tourette's syndrome. Neuromodulation. (2013) 16:55–8. 10.1111/j.1525-1403.2012.00526.x 23240689

[77] Dehning S Leitner B Schennach R Muller N Botzel K Obermeier M . Functional outcome and quality of life in Tourette's syndrome after deep brain stimulation of the posteroventrolateral globus pallidus internus: long-term follow-up. World J Biol Psychiatry. (2014) 15:66–75. 10.3109/15622975.2013.849004 24304122

[78] Dong S Zhang X Li J Li Y. The benefits of low-frequency pallidal deep brain stimulation in a patient with Tourette syndrome. Parkinsonism Relat Disord. (2014) 20:1438–9. 10.1016/j.parkreldis.2014.09.028 25306201

[79] Huasen B McCreary R Evans J Potter G Silverdale M. Cervical myelopathy secondary to Tourette's syndrome managed by urgent deep brain stimulation. Mov Disord. (2014) 29:452–3. 10.1002/mds.25797 24395181

[80] Patel N Jimenez-Shahed J. Simultaneous improvement of tics and parkinsonism after pallidal DBS. Parkinsonism Relat Disord. (2014) 20:1022–3. 10.1016/j.parkreldis.2014.05.009 24957594

[81] Pourfar MH Budman CL Mogilner AY. A case of deep brain stimulation for Tourette's complicated by twiddler's syndrome. Mov Disord Clin Pract. (2015) 2:192–3. 10.1002/mdc3.12132 30713894 PMC6353532

[82] Sachdev PS Mohan A Cannon E Crawford JD Silberstein P Cook R . Deep brain stimulation of the antero-medial globus pallidus interna for Tourette syndrome. PLoS ONE. (2014) 9:e104926. 10.1371/journal.pone.0104926 25136825 PMC4138156

[83] Cannon E Silburn P Coyne T O'Maley K Crawford JD Sachdev PS. Deep brain stimulation of anteromedial globus pallidus interna for severe Tourette's syndrome. Am J Psychiatry. (2012) 169:860–6. 10.1176/appi.ajp.2012.11101583 22772329

[84] Zhang JG Ge Y Stead M Zhang K Yan SS Hu W . Long-term outcome of globus pallidus internus deep brain stimulation in patients with Tourette syndrome. Mayo Clin Proc. (2014) 89:1506–14. 10.1016/j.mayocp.2014.05.019 25444487

[85] Kefalopoulou Z Zrinzo L Jahanshahi M Candelario J Milabo C Beigi M . Bilateral globus pallidus stimulation for severe Tourette's syndrome: a double-blind, randomised crossover trial. Lancet Neurol. (2015) 14:595–605. 10.1016/S1474-4422(15)00008-3 25882029

[86] Morreale F Kefalopoulou Z Zrinzo L Limousin P Joyce E Foltynie T . Inhibitory control on a stop signal task in Tourette syndrome before and after deep brain stimulation of the internal segment of the globus pallidus. Brain Sci. (2021) 11:461. 10.3390/brainsci11040461 33916444 PMC8066761

[87] Wardell K Kefalopoulou Z Diczfalusy E Andersson M Astrom M Limousin P . Deep brain stimulation of the pallidum internum for Gilles de la Tourette syndrome: a patient-specific model-based simulation study of the electric field. Neuromodulation. (2015) 18:90–6. 10.1111/ner.12248 25284508

[88] Cury RG Lopez WO Dos Santos Ghilardi MG Barbosa DC Barbosa ER Teixeira MJ . Parallel improvement in anxiety and tics after DBS for medically intractable Tourette syndrome: a long-term follow-up. Clin Neurol Neurosurg. (2016) 144:33–5. 10.1016/j.clineuro.2016.02.030 26963088

[89] Huys D Bartsch C Koester P Lenartz D Maarouf M Daumann J . Motor improvement and emotional stabilization in patients with Tourette syndrome after deep brain stimulation of the ventral anterior and ventrolateral motor part of the thalamus. Biol Psychiatry. (2016) 79:392–401. 10.1016/j.biopsych.2014.05.014 25034948

[90] Smeets A Duits AA Plantinga BR Leentjens AFG Oosterloo M Visser-Vandewalle V . Deep brain stimulation of the internal globus pallidus in refractory Tourette syndrome. Clin Neurol Neurosurg. (2016) 142:54–9. 10.1016/j.clineuro.2016.01.020 26811866

[91] Testini P Zhao CZ Stead M Duffy PS Klassen BT Lee KH. Centromedian-Parafascicular complex deep brain stimulation for Tourette syndrome: a retrospective study. Mayo Clin Proc. (2016) 91:218–25. 10.1016/j.mayocp.2015.11.016 26848003 PMC4765735

[92] Zhang XH Li JY Zhang YQ Li YJ. Deep brain stimulation of the globus pallidus internus in patients with intractable Tourette syndrome: a 1-year follow-up study. Chin Med J. (2016) 129:1022–7. 10.4103/0366-6999.180512 27098785 PMC4852667

[93] Akbarian-Tefaghi L Akram H Johansson J Zrinzo L Kefalopoulou Z Limousin P . Refining the deep brain stimulation target within the limbic globus pallidus internus for Tourette syndrome. Stereotact Funct Neurosurg. (2017) 95:251–8. 10.1159/000478273 28787721

[94] Dwarakanath S Hegde A Ketan J Chandrajit P Yadav R Keshav K . “I swear, I can't stop it!” - a case of severe Tourette's syndrome treated with deep brain stimulation of anteromedial globus pallidus interna. Neurol India. (2017) 65:99–102. 10.4103/0028-3886.198188 28084249

[95] Neudorfer C El Majdoub F Hunsche S Richter K Sturm V Maarouf M. Deep Brain stimulation of the H fields of forel alleviates tics in Tourette syndrome. Front Hum Neurosci. (2017) 11:308. 10.3389/fnhum.2017.00308 28659777 PMC5468420

[96] Picillo M Rohani M Lozano AM Fasano A. Two indications, one target: concomitant epilepsy and tourettism treated with centromedian/parafascicular thalamic stimulation. Brain Stimul. (2017) 10:711–3. 10.1016/j.brs.2017.01.577 28117177

[97] Welter M-L Houeto J-L Thobois S Bataille B Guenot M Worbe Y . Anterior pallidal deep brain stimulation for Tourette's syndrome: a randomised, double-blind, controlled trial. Lancet Neurol. (2017) 16:610–9. 10.1016/S1474-4422(17)30160-6 28645853

[98] Azimi A Parvaresh M Shahidi G Habibi A Rohani S Safdarian M . Anteromedial GPi deep brain stimulation in Tourette syndrome: the first case series from Iran. Clin Neurol Neurosurg. (2018) 172:116–9. 10.1016/j.clineuro.2018.06.045 29990958

[99] Doshi PK Ramdasi R Thorve S. Deep brain stimulation of anteromedial globus pallidus internus for severe Tourette syndrome. Indian J Psychiatry. (2018) 60:138–40. 10.4103/psychiatry.IndianJPsychiatry_53_18 29736078 PMC5914244

[100] Dowd RS Pourfar M Mogilner AY. Deep brain stimulation for Tourette syndrome: a single-center series. J Neurosurg. (2018) 128:596–604. 10.3171/2016.10.JNS161573 28387621

[101] Kano Y Matsuda N Nonaka M Fujio M Kono T Kaido T. Sensory phenomena and obsessive-compulsive symptoms in Tourette syndrome following deep brain stimulation: two case reports. J Clin Neurosci. (2018) 56:199–201. 10.1016/j.jocn.2018.06.046 30042071

[102] Richieri R Blackman G Musil R Spatola G Cavanna AE Lancon C . Positive clinical effects of gamma knife capsulotomy in a patient with deep brain stimulation-refractory Tourette syndrome and obsessive compulsive disorder. Clin Neurol Neurosurg. (2018) 170:34–7. 10.1016/j.clineuro.2018.04.018 29723733

[103] Brito M Teixeira MJ Mendes MM Franca C Iglesio R Barbosa ER . Exploring the clinical outcomes after deep brain stimulation in Tourette syndrome. J Neurol Sci. (2019) 402:48–51. 10.1016/j.jns.2019.05.011 31103958

[104] Kakusa B Saluja S Tate WJ Espil FM Halpern CH Williams NR. Robust clinical benefit of multi-target deep brain stimulation for treatment of Gilles de la Tourette syndrome and its comorbidities. Brain Stimul. (2019) 12:816–8. 10.1016/j.brs.2019.02.026 30878341

[105] Rossi M Cerquetti D Cammarota A Merello M. Tourette syndrome: clinical benefit with unilateral stimulation after bilateral pallidal implant. Mov Disord. (2019) 34:580–2. 10.1002/mds.27636 30801769

[106] Zhang C Li H Pan Y Jin H Sun B Wu Y . Pallidal neurostimulation and capsulotomy for malignant Tourette's syndrome. Mov Disord Clin Pract. (2019) 6:393–5. 10.1002/mdc3.12761 31286009 PMC6592792

[107] Zhang C Deng Z Pan Y Zhang J Zeljic K Jin H . Pallidal deep brain stimulation combined with capsulotomy for Tourette's syndrome with psychiatric comorbidity. J Neurosurg. (2019) 131:1788–96. 10.3171/2018.8.JNS181339 30611137

[108] Zhu GY Geng XY Zhang RL Chen YC Liu YY Wang SY . Deep brain stimulation modulates pallidal and subthalamic neural oscillations in Tourette's syndrome. Brain Behav. (2019) 9:e01450. 10.1002/brb3.1450 31647199 PMC6908859

[109] Duarte-Batista P Coelho M Quintas S Levy P Castro Caldas A Goncalves-Ferreira A . Anterior limb of internal capsule and bed nucleus of stria terminalis stimulation for Gilles de la Tourette syndrome with obsessive-compulsive disorder in adolescence: a case of success. Stereotact Funct Neurosurg. (2020) 98:95–103. 10.1159/000505702 32209787

[110] Servello D Zekaj E Saleh C Lange N Porta M. Deep brain stimulation in Gilles de la Tourette syndrome: what does the future hold? A cohort of 48 patients. Neurosurgery. (2016) 78:91–100. 10.1227/NEU.0000000000001004 26348012

[111] Servello D Porta M Sassi M Brambilla A Robertson MM. Deep brain stimulation in 18 patients with severe Gilles de la Tourette syndrome refractory to treatment: the surgery and stimulation. J Neurol Neurosurg Psychiatry. (2008) 79:136–42. 10.1136/jnnp.2006.104067 17846115

[112] Porta M Brambilla A Cavanna AE Servello D Sassi M Rickards H . Thalamic deep brain stimulation for treatment-refractory Tourette syndrome: two-year outcome. Neurology. (2009) 73:1375–80. 10.1212/WNL.0b013e3181bd809b 19858459

[113] Porta M Servello D Zanaboni C Anasetti F Menghetti C Sassi M . Deep brain stimulation for treatment of refractory Tourette syndrome: long-term follow-up. Acta Neurochir. (2012) 154:2029–41. 10.1007/s00701-012-1497-8 22961243

[114] Marceglia S Prenassi M Galbiati TF Porta M Zekaj E Priori A . Thalamic local field potentials are related to long-term DBS effects in Tourette syndrome. Front Neurol. (2021) 12:578324. 10.3389/fneur.2021.578324 33658970 PMC7917178

[115] Andrade P Heiden P Hoevels M Schlamann M Baldermann JC Huys D . Modulation of fibers to motor cortex during thalamic DBS in Tourette patients correlates with tic reduction. Brain Sci. (2020) 10. 10.3390/brainsci10050302 32429216 PMC7287978

[116] Kimura Y Iijima K Takayama Y Yokosako S Kaneko Y Omori M . Deep brain stimulation for refractory Tourette syndrome: electrode position and clinical outcome. Neurol Med Chir. (2021) 61:33–9. 10.2176/nmc.oa.2020-0202 33239475 PMC7812307

[117] Müller-Vahl KR Szejko N Saryyeva A Schrader C Krueger D Horn A . Randomized double-blind sham-controlled trial of thalamic versus GPi stimulation in patients with severe medically refractory Gilles de la Tourette syndrome. Brain Stimul. (2021) 14:662–75. 10.1016/j.brs.2021.04.004 33857664

[118] Sun F Zhang X Dong S Zhang Y Li J Wang Y . Effectiveness of low-frequency pallidal deep brain stimulation at 65 hz in Tourette syndrome. Neuromodulation. (2021). 10.1111/ner.13456 [Epub ahead of print]. 35125148

[119] Baldermann JC Kuhn J Schüller T Kohl S Andrade P Schleyken S . Thalamic deep brain stimulation for Tourette syndrome: a naturalistic trial with brief randomized, double-blinded sham-controlled periods. Brain Stimul. (2021) 14:1055–418. 10.1016/j.brs.2021.07.003 34245918

[120] Ewert S Plettig P Li N Chakravarty MM Collins DL Herrington TM . Toward defining deep brain stimulation targets in MNI space: a subcortical atlas based on multimodal MRI, histology and structural connectivity. Neuroimage. (2018) 170:271–82. 10.1016/j.neuroimage.2017.05.015 28536045

[121] Xiao Y Fonov V Chakravarty MM Beriault S Al Subaie F Sadikot A . A dataset of multi-contrast population-averaged brain MRI atlases of a Parkinson's disease cohort. Data Brief. (2017) 12:370–9. 10.1016/j.dib.2017.04.013 28491942 PMC5413210

[122] Fayad SM Guzick AG Reid AM Mason DM Bertone A Foote KD . Six-nine year follow-up of deep brain stimulation for obsessive-compulsive disorder. PLoS ONE. (2016) 11:e0167875. 10.1371/journal.pone.0167875 27930748 PMC5145226

[123] Mahajan UV Purger DA Mantovani A Williams NR Espil FM Han SS . Deep brain stimulation results in greater symptomatic improvement in Tourette syndrome than conservative measures: a meta-analysis. Stereotact Funct Neurosurg. (2020) 98:270–7. 10.1159/000507059 32434201

[124] Hirschtritt ME Lee PC Pauls DL Dion Y Grados MA Illmann C . Lifetime prevalence, age of risk, and genetic relationships of comorbid psychiatric disorders in Tourette syndrome. JAMA Psychiatry. (2015) 72:325–33. 10.1001/jamapsychiatry.2014.2650 25671412 PMC4446055

[125] Macchi G Jones EG. Toward an agreement on terminology of nuclear and subnuclear divisions of the motor thalamus. J Neurosurg. (1997) 86:670–85. 10.3171/jns.1997.86.4.0670 9120632

[126] Mai JK Majtanik M. Toward a common terminology for the thalamus. Front Neuroanat. (2019) 12:114. 10.3389/fnana.2018.00114 30687023 PMC6336698

[127] Visser-Vandewalle V Temel Y Boon P Vreeling F Colle H Hoogland G . Chronic bilateral thalamic stimulation: a new therapeutic approach in intractable Tourette syndrome: report of three cases. J Neurosurg. (2003) 99:1094–100. 10.3171/jns.2003.99.6.1094 14705742

[128] Williams NR Taylor JJ Lamb K Hanlon CA Short EB George MS. Role of functional imaging in the development and refinement of invasive neuromodulation for psychiatric disorders. World J Radiol. (2014) 6:756–78. 10.4329/wjr.v6.i10.756 25349661 PMC4209423

[129] Krauss JK Yianni J Loher TJ Aziz TZ. Deep brain stimulation for dystonia. J Clin Neurophysiol. (2004) 21:18–30. 10.1097/00004691-200401000-00004 15097291

[130] Yianni J Bain P Giladi N Auca M Gregory R Joint C . Globus pallidus internus deep brain stimulation for dystonic conditions: a prospective audit. Mov Disord. (2003) 18:436–42. 10.1002/mds.10380 12671953

[131] Johnson KA Duffley G Foltynie T Hariz M Zrinzo L Joyce EM . Basal ganglia pathways associated with therapeutic pallidal deep brain stimulation for Tourette syndrome. Biol Psychiatry Cogn Neurosci Neuroimaging. (2020). 10.1016/j.bpsc.2020.11.005 [Epub ahead of print]. 33536144 PMC8864935

[132] Szejko N Müller-Vahl KR. Challenges in the diagnosis and assessment in patients with Tourette syndrome and comorbid obsessive-compulsive disorder. Neuropsychiatr Dis Treat. (2021) 17:1253–66. 10.2147/NDT.S251499 33958867 PMC8096634

[133] Cath DC Spinhoven P Hoogduin CAL Landman AD van Woerkom TCAM van de Wetering BJM . Repetitive behaviors in Tourette's syndrome and OCD with and without tics: what are the differences? Psychiatry Res. (2001) 101:171–85. 10.1016/S0165-1781(01)00219-0 11286820

[134] Ganos C Kahl U Brandt V Schunke O Baumer T Thomalla G . The neural correlates of tic inhibition in Gilles de la Tourette syndrome. Neuropsychologia. (2014) 65:297–301. 10.1016/j.neuropsychologia.2014.08.007 25128587

[135] van der Salm SMA van der Meer JN Cath DC Groot PFC van der Werf YD Brouwers E . Distinctive tics suppression network in Gilles de la Tourette syndrome distinguished from suppression of natural urges using multimodal imaging. Neuroimage Clin. (2018) 20:783–92. 10.1016/j.nicl.2018.09.014 30268027 PMC6169325

[136] Schrock LE Mink JW Woods DW Porta M Servello D Visser-Vandewalle V . Tourette syndrome deep brain stimulation: a review and updated recommendations. Mov Disord. (2015) 30:448–71. 10.1002/mds.26094 25476818

[137] Welter ML Mallet L Houeto JL Karachi C Czernecki V Cornu P . Internal pallidal and thalamic stimulation in patients with Tourette syndrome. Arch Neurol. (2008) 65:952–7. 10.1001/archneur.65.7.952 18625864

[138] Van der Linden C Colle H Vandewalle V Alessi G Rijckaert D De Waele L. Successful treatment of tics with bilateral internal pallidum (GPi) stimulation in a 27-year-old male patient with Gilles de la Tourette's syndrome (GTS). Mov Disord. (2002) 17:S341–S.

[139] Ackermans L Duits A Temel Y Winogrodzka A Peeters F Beuls EA . Long-term outcome of thalamic deep brain stimulation in two patients with Tourette syndrome. J Neurol Neurosurg Psychiatry. (2010) 81:1068–72. 10.1136/jnnp.2009.176859 20660922

[140] Servello D Saleh C Bona AR Zekaj E Porta M. After 19 years of deep brain stimulation in Tourette's syndrome: from multiple targets to one single target? Surg Neurol Int. (2018) 9:219. 10.4103/sni.sni_271_18 30505621 PMC6219290

[141] Middlebrooks EH Tuna I Grewal S Almeida L Heckman M Lesser E . Segmentation of the globus pallidus internus using probabilistic diffusion tractography for deep brain stimulation targeting in Parkinson disease. Am J Neuroradiol. (2018) 39:1127–34. 10.3174/ajnr.A5641 29700048 PMC7410622

[142] Horn A Fox MD. Opportunities of connectomic neuromodulation. Neuroimage. (2020) 221:117180. 10.1016/j.neuroimage.2020.117180 32702488 PMC7847552

[143] Paulo DL Bick SK. Advanced imaging in psychiatric neurosurgery: toward personalized treatment. Neuromodulation. (2021). 10.1111/ner.13392 [Epub ahead of print]. 35125138

[144] Kakusa B Saluja S Barbosa DAN Cartmell S Espil FM Williams NR . Evidence for the role of the dorsal ventral lateral posterior thalamic nucleus connectivity in deep brain stimulation for Gilles de la Tourette syndrome. J Psychiatr Res. (2021) 132:60–4. 10.1016/j.jpsychires.2020.09.024 33045620

[145] Johnson KA Fletcher PT Servello D Bona A Porta M Ostrem JL . Image-based analysis and long-term clinical outcomes of deep brain stimulation for Tourette syndrome: a multisite study. J Neurol Neurosurg Psychiatry. (2019) 90:1078–90. 10.1136/jnnp-2019-320379 31129620 PMC6744301

[146] Deeb W Rossi PJ Porta M Visser-Vandewalle V Servello D Silburn P . The international deep brain stimulation registry and database for Gilles de la Tourette syndrome: how does it work? Front Neurosci. (2016) 10:170. 10.3389/fnins.2016.00170 27199634 PMC4842757

[147] Baumung L Müller-Vahl K Dyke K Jackson G Jackson S Golm D . Developing the premonitory urges for tic disorders scale–revised (PUTS-R). J Neuropsychol. (2021) 15:129–42. 10.1111/jnp.12216 32543110

[148] Cavanna AE Schrag A Morley D Orth M Robertson MM Joyce E . The Gilles de la Tourette syndrome–quality of life scale (GTS-QOL): development and validation. J Neurol. (2008) 71:1410–6. 10.1212/01.wnl.0000327890.02893.61 18955683

[149] McGuinness LA Higgins JPT. Risk-of-bias VISualization (robvis): an R package and shiny web app for visualizing risk-of-bias assessments. Res Synth Methods. (2020) 12:55–61. 10.1002/jrsm.1411 32336025

