# Supplementary figures and images for: Target-Specific Effects of Deep Brain Stimulation for Tourette Syndrome: A Systematic Review and Meta-Analysis
Source: Front Neurol. 2021 Oct 20;12:769275. doi: 10.3389/fneur.2021.769275 (PMC8563609; doi:10.3389/fneur.2021.769275)

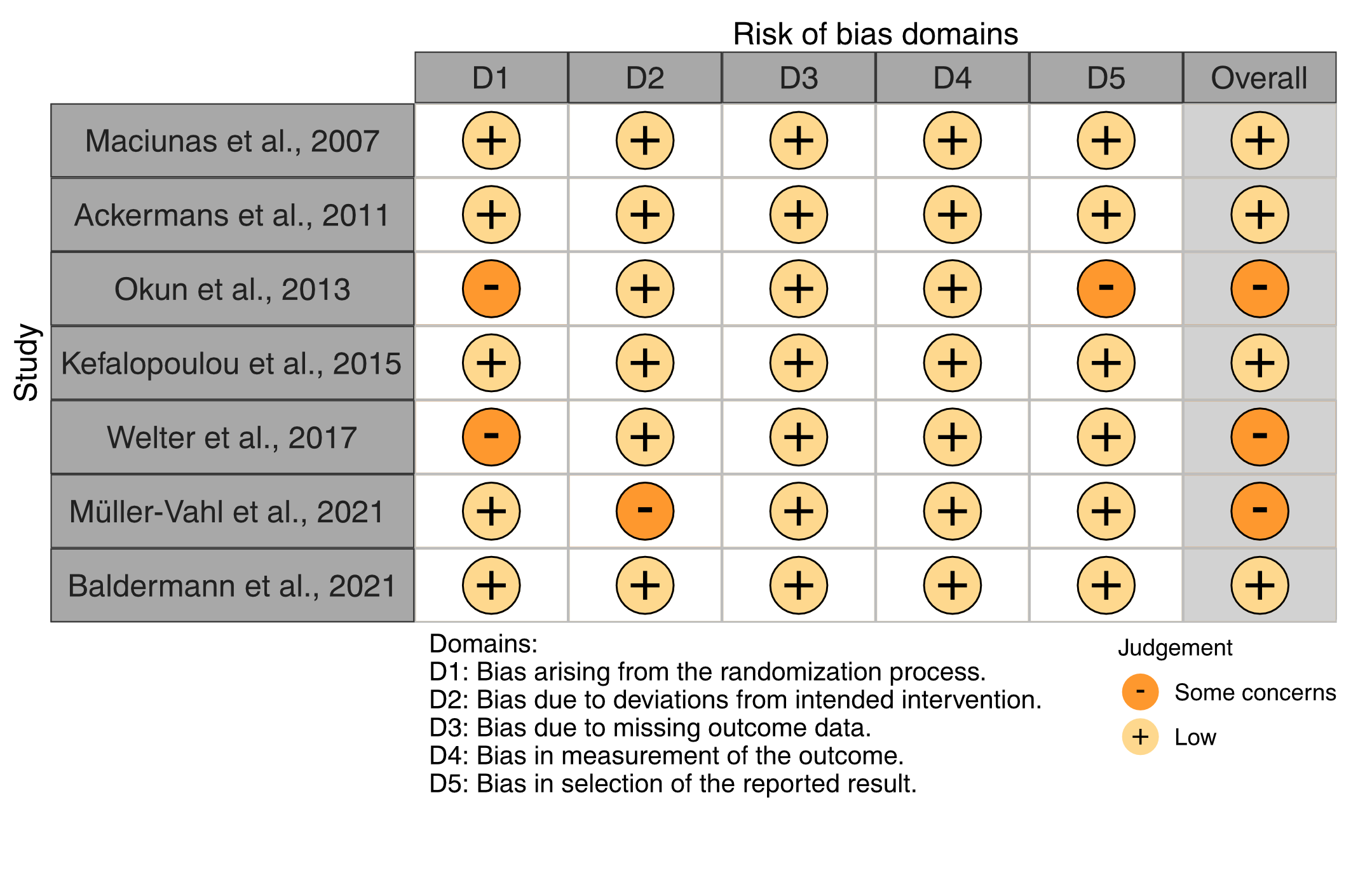

Supplement: Supplementary Figure 1 — Summary table of risk of bias domains in each RCT created with the risk of bias visualization (robvis) tool (149). [file Image_1.TIFF]
