# Supplementary material for: Target-Specific Effects of Deep Brain Stimulation for Tourette Syndrome: A Systematic Review and Meta-Analysis
Source: Front Neurol. 2021 Oct 20;12:769275. doi: 10.3389/fneur.2021.769275 (PMC8563609; doi:10.3389/fneur.2021.769275)
Supplement: Supplementary Material 1 — Search terms. [file Table_1.DOCX]

Supplement Materials 1 Search Terms:

| **Database** | **Search Syntax** |
| --- | --- |
| **Pubmed** | (("tourette syndrome"[MeSH Terms] OR ("tourette"[All Fields] AND "syndrome"[All Fields]) OR "tourette syndrome"[All Fields] OR ("tourette syndrome"[MeSH Terms] OR ("tourette"[All Fields] AND "syndrome"[All Fields]) OR "tourette syndrome"[All Fields] OR ("gilles"[All Fields] AND "de"[All Fields] AND "la"[All Fields] AND "tourette"[All Fields] AND "syndrome"[All Fields]) OR "gilles de la tourette syndrome"[All Fields]) OR ("tourette syndrome"[MeSH Terms] OR ("tourette"[All Fields] AND "syndrome"[All Fields]) OR "tourette syndrome"[All Fields] OR ("tourettes"[All Fields] AND "disorder"[All Fields]) OR "tourettes disorder"[All Fields]) OR ("tic disorders"[MeSH Terms] OR ("tic"[All Fields] AND "disorders"[All Fields]) OR "tic disorders"[All Fields] OR ("tic"[All Fields] AND "disorder"[All Fields]) OR "tic disorder"[All Fields])) AND ("deep brain stimulation"[MeSH Terms] OR ("deep"[All Fields] AND "brain"[All Fields] AND "stimulation"[All Fields]) OR "deep brain stimulation"[All Fields] OR "DBS"[All Fields])) AND (1999/1/1:2021/7/8[pdat]) |
